# Nanocomposite Hydrogels: A Promising Approach for the Treatment of Degenerative Joint Diseases

**DOI:** 10.1002/smsc.202400236

**Published:** 2024-09-03

**Authors:** Qizhu Chen, Zitian Zheng, Mian Lin, Zhengyu Guo, Hongjie Huang, Qingyun Xue, Shengdan Jiang, Jianquan Wang, Aimin Wu

**Affiliations:** ^1^ Department of Orthopaedics Key Laboratory of Structural Malformations in Children of Zhejiang Province Key Laboratory of Orthopaedics of Zhejiang Province The Second Affiliated Hospital and Yuying Children's Hospital of Wenzhou Medical University Wenzhou Zhejiang 325000 China; ^2^ Department of Clinic of Spine Center Xinhua Hospital Shanghai Jiaotong University School of Medicine Shanghai 200082 China; ^3^ Department of Sports Medicine, Peking University Third Hospital Institute of Sports Medicine of Peking University; Beijing Key Laboratory of Sports Injuries, Engineering Research Center of Sports Trauma Treatment Technology and Devices, Ministry of Education Beijing 100191 P. R. China; ^4^ Department of Orthopedics Beijing Hospital National Center of Gerontology Institute of Geriatric Medicine Chinese Academy of Medical Sciences Beijing 100730 P. R. China; ^5^ Department of Orthopaedic Surgery The Third Affiliated Hospital of Wenzhou Medical University 108 WanSong Road, Ruian Wenzhou Zhejiang 325200 China

**Keywords:** bone tissue engineering applications, cartilage defects, degenerative joint diseases, intervertebral disc degenerations, nanocomposite hydrogels, osteoarthritis

## Abstract

Degenerative joint diseases, as a global public health issue, impose significant burdens on patients’ lives and substantial economic costs on society. Currently, the primary modalities include physical therapy, pharmaceutical intervention, and surgical procedures. None of these approaches can alter the course of this degenerative process. Due to their commendable biocompatibility, biodegradability, and heightened efficacy in drug delivery, hydrogels present themselves as a novel noninvasive remedy for degenerative joint ailments. However, the clinical application of hydrogels still faces some challenges, including the uncontrolled discharge of encapsulated medications, the absence of adequate mechanical reinforcement for destabilized joints, and adaptability to fluctuating microenvironments. Recently, nanocomposite hydrogels, formed by introducing nanomaterials into hydrogels by physical or chemical means, can improve the limitations of hydrogels and extend their potential for biological applications in degenerative joint diseases. In this study, the pathologic features of degenerative joint diseases and the multiple applications of different types of nanocomposite hydrogels in targeting these different pathologic features are briefly described. It also concludes with an outlook on the use of nanocomposite hydrogels in clinical settings and discusses their challenges and limitations.

## Graphical abstract

By combining various nanomaterials with hydrogels, multifunctional nanocomposite hydrogels are developed for treating degenerative joint diseases. In osteoarthritis, they provide lubrication and anti‐inflammatory effects; in cartilage defect, they adhere to and repair damaged cartilage, promoting regeneration; in degenerative intervertebral disc, they offer mechanical support and recruit stem cells to facilitate nucleus pulposus regeneration.

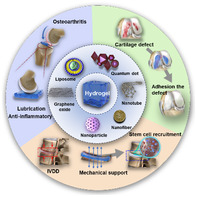

## 1 Introduction

Degenerative joint diseases constitute a class of disorders characterized by continual deterioration and progression. These encompass intervertebral disc degeneration (IVDD), osteoarthritis (OA), and cartilage defects. Individuals afflicted with these conditions experience severe limitations in mobility and flexibility, leading to premature retirement, diminished well‐being, and reduced societal engagement. Moreover, the treatment of these conditions significantly amplifies medical costs for both society and patients’ families.^[^ ^1^ ^]^ Currently, conservative and surgical interventions constitute the primary modalities in the clinical management of degenerative joint diseases. While conservative approaches primarily target symptom alleviation rather than reversing joint degeneration or restoring original function, they are accompanied by the potential for numerous side effects due to prolonged medication. Additionally, surgical intervention poses inherent risks to patients, including trauma, high procedural risks, and substantial financial burdens. Consequently, there is a pressing need for the development of noninvasive, nonsurgical treatments for degenerative joint diseases.

In recent years, extensive research has been conducted on various biocompatible materials suitable for application in degenerative joint conditions, including nanoparticles (NPs), microneedles, microspheres, liposomes, and hydrogels^[^ ^2^ ^]^ Hydrogels, characterized by their hydrophilic soft polymer composition and 3D network structure, hold significant promise in delaying IVDD and facilitating articular cartilage repair due to their unique mechanical properties, physicochemical attributes, and biocompatibility.^[^ ^3^ ^]^ Research indicates that hydrogels possess intricate pore/channel structures, rendering them conducive to moisture absorption, drug delivery, and cell culture applications. They provide a spatially conducive environment for 3D cell growth and facilitate the complete release of administered drugs.^[^ ^4^ ^]^ However, current hydrogel‐based systems encounter limitations such as restricted mechanical properties, inconsistent drug release kinetics, and inadequate responsiveness to environmental cues, thereby limiting their utility in degenerative joint diseases.[^4^, ^5^]

Advancements in nanotechnology have revealed the potential of nanomaterials in addressing degenerative joint ailments.^[^ ^6^ ^]^ The distinct size and physicochemical properties of nanomaterials facilitate their penetration through the extracellular matrix (ECM) barrier and internalization into diverse cell types’ cytoplasm.^[^ ^7^ ^]^ Nanomaterials encompass organic and inorganic variants. Inorganic nanomaterials encompass oxide nanomaterials,^[^ ^8^ ^]^ metal nanomaterials,^[^ ^9^ ^]^ quantum dots,^[^ ^10^ ^]^ capable of adsorbing and loading drugs onto their functional groups, and porous structures. Conversely, organic nanomaterials such as micelles,^[^ ^11^ ^]^ liposomes,^[^ ^12^ ^]^ and proteins,^[^ ^13^ ^]^ owing to their chemical versatility, can be tailored for responsive properties like pH, temperature, reactive oxygen species (ROS), specific protease, and mechanical stress responses. Nevertheless, despite their advantageous characteristics, nanomaterials suffer from limitations, including inadequate mechanical support provision within joints, narrow treatment windows during joint fluid metabolism, and lack of stability and long‐term efficacy.

Impressively, numerous studies have shown that nanomaterials can be incorporated into hydrogel networks through chemical bonding or physical adsorption to form nanocomposite hydrogels. The combination of hydrogels and nanomaterials can integrate the advantages of both biomaterials and circumvent the disadvantages of each.^[^ ^14^ ^]^ Compared with ordinary hydrogels, the incorporation of nanomaterials can be used as the active ingredients of nanocomposite hydrogels, providing hydrogels with some new properties.^[^ ^15^ ^]^ For instance, nanocomposite hydrogels serve as a carrier, offering a stable loading platform for the bioactive components within the hydrogel, thereby extending the residence time of the cargo within the joint, enhancing the bioavailability of the cargo within the joint, and improving its distribution within the articular cavity.^[^ ^16^ ^]^ Furthermore, the incorporation of nanomaterials endows nanocomposite hydrogels with the capability to respond to the degenerative microenvironment within joints, facilitating the tailored biodegradable release of cargo.^[^ ^17^ ^]^ More significantly, the incorporation of nanomaterials into hydrogels through various methods such as overall assembly, hydrogen bonding, chemical bonding, electrostatic adsorption, and physical adsorption can significantly enhance the mechanical properties of nanocomposite hydrogels.^[^ ^18^ ^]^ This enhancement effectively compensates for the loss of mechanical characteristics in joints during degeneration, such as lubrication,^[15c]^ elasticity,^[^ ^19^ ^]^ and torsional resistance.^[^ ^20^ ^]^

**Scheme 1:**
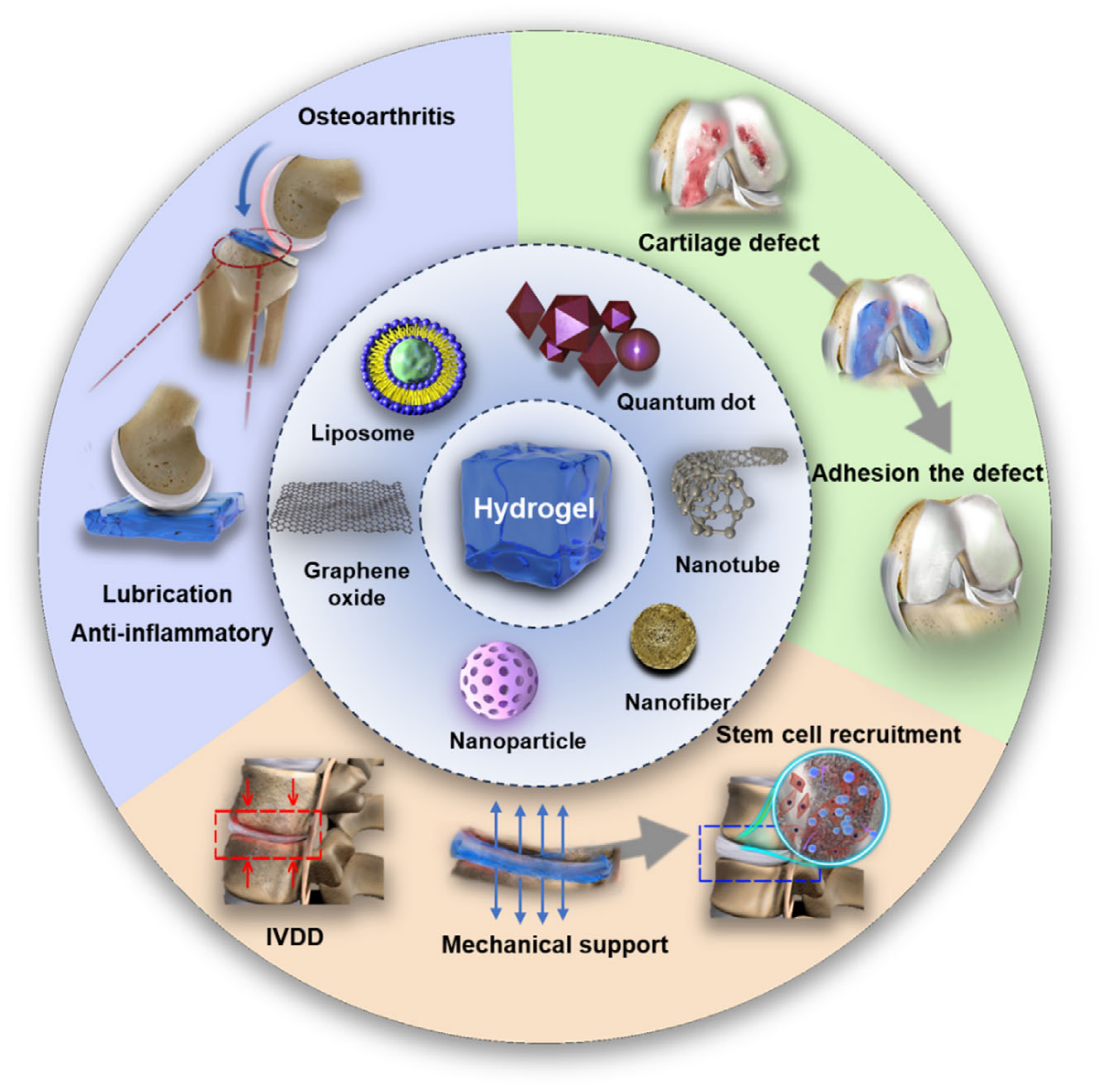
Specialized treatments with nanocomposite hydrogel for various degenerative joint diseases.

To date, despite considerable research and reviews on hydrogels, there remains a lack of a comprehensive, detailed review addressing the application of nanohydrogels in treating degenerative joint diseases. In this review, we comprehensively present and discuss the application of nanocomposite hydrogels in degenerative joint diseases, focusing on their pathogenetic characteristics (**Scheme** **1**). We provide a detailed comparison and summary of the advantages of different bio‐nanomaterials integrated with various hydrogels in treating degenerative joint diseases, which include specific mechanical properties, excellent anti‐inflammatory capacity, induction of stem cell differentiation, smart‐response release, and other functionalities. Finally, we discuss the potential of nanocomposite hydrogels in clinical applications, as well as their associated challenges and limitations.

## 2 Nanocomposite Hydrogels: Composition and Properties

### 2.1 Definition of Nanocomposite Hydrogels

Nanocomposite hydrogels are a class of hybrid biomaterials that integrate nanoscale components, such as NPs, nanofibers, or nanosheets, into a hydrogel matrix.^[^ ^21^ ^]^ These nanomaterials can be made from various materials, including polymers, ceramics, metals, or carbon‐based structures, and are typically in the size range of 1–100 nm.^[^ ^22^ ^]^ The incorporation of nanomaterials into hydrogels can significantly enhance their mechanical, physicochemical, and biological properties, making them suitable for a wide range of biomedical applications, particularly in bone tissue engineering.^[^ ^23^ ^]^

One of the key advantages of nanocomposite hydrogels is their ability to mimic the hierarchical structure and biochemical composition of the native ECM.^[^ ^24^ ^]^ By carefully designing the hydrogel matrix and selecting appropriate nanomaterials, researchers can create biomimetic scaffolds that closely resemble the tissue‐specific microenvironment, facilitating cell adhesion, proliferation, and differentiation.^[^ ^25^ ^]^ Moreover, the high surface area‐to‐volume ratio of nanomaterials can enhance cell‐material interactions and provide a larger platform for the immobilization of bioactive molecules, such as growth factors or cell‐adhesive peptides.^[^ ^26^ ^]^ Combining hydrogels and nanomaterials into nanocomposite hydrogels can further enhance these benefits and circumvent their disadvantages. The nanocomposite hydrogel formulation can offer improved mechanical properties and structural stability, addressing the limitations of hydrogels alone, while retaining the biocompatibility and bioactivity of the nanomaterials. Additionally, the synergistic effects of combining these materials can lead to enhanced therapeutic outcomes, making nanocomposite hydrogels a promising approach for bone tissue engineering applications. It is important to emphasize that during the incorporation of nanomaterials into the hydrogel matrix, the uniform distribution of the nanomaterials must be ensured to prevent aggregation, which could result in heterogeneous properties and diminished functionality.^[^ ^27^ ^]^ In the context of bone tissue engineering, nanocomposite hydrogels have been extensively investigated for the repair and regeneration of various musculoskeletal tissues, including articular cartilage,^[^ ^28^ ^]^ bone,^[^ ^29^ ^]^ and intervertebral discs (IVDs).^[^ ^30^ ^]^ The combination of hydrogels and nanomaterials into nanocomposite hydrogels represents a cutting‐edge approach in the field, offering the potential to overcome current limitations and significantly improve the outcomes of tissue engineering strategies. The combination of various hydrogels with different nanomaterials to form nanocomposite hydrogels represents a cutting‐edge approach in the field, offering the potential to overcome current limitations.

### 2.2 Components of Nanocomposite Hydrogels

Nanocomposite hydrogels are composed of two main components: a polymer matrix and NPs or nanofibers. The careful selection and combination of these components allow researchers to tailor the properties of the resulting nanocomposite hydrogel to suit specific applications in bone tissue engineering (**Table** **1**).

**Table 1:** Nanocomposite hydrogels for cartilage defect, OA, and IVDD.

Hydrogels matrix	Hydrogel characteristic	Nanomaterials	application	property of therapeutic	References
HA	Enhancing mechanical properties	Cellulose nanofibril CNF	Cartilage defect	Inducting MSCs differentiation	[133]
Alginate/gelatin/chondroitin sulfate	Excellent biocompatibility and processability	GO	Cartilage defect	Printability and inducting ADSCs differentiation	[90]
Aldehyde polyethylene glycol/carboxymethyl chitosan (APA/CMC)	Enhancing mechanical properties, injectable, inspired by tunnel‐piled structure of subway tunnels	Thermosensitive gelatin microrods (GMs) and coaxial electrospun polylactic acid/gelatin fibers (PGFs) loaded with KGN	Cartilage defect	Good self‐healing ability, improved mechanical properties, sustained KGN release; promotes BMSC proliferation and ingrowth; enhances chondrogenesis and neocartilage formation	[131]
Alginate‐based micro‐cavity hydrogel (MCG)	Excellent biocompatibility and processability	miPSC‐seeded constructs	Cartilage defect	Inducting iPSCs differentiation	[136]
Star acrylate‐terminated lactide‐chain‐extended polyethylene glycol macromer	Tunable modulus and degradation	Hydroxyapatite	Cartilage defect	Delivery cytokine and enhancing hMSCs differentiation	[139]
Alginate	Accelerating mineralization of engineered cartilage templates	α‐tricalcium phosphate (α‐TCP) nanoparticle	Cartilage defect	Delivery cytokine and promoting intrachondral bone formation	[141]
PVA	High water content, high water absorption, and high elasticity	Fe_2_O_3_ nanoparticles	Cartilage defect	Mechanical support and inducing BMSCs differentiation	[130a]
Two‐component bioinstructive matrix (VitroGel‐RGD)	High piezoelectricity and high biocompatibility	GO nanosheets	Cartilage defect	Enhancing the mechanical strength, electrical conductivity, and bioactivity	[146]
CMCht‐SF	Comparable water content	Wet‐electrospun poly(3‐hydroxybutyrate*‐co*‐3‐hydroxyvalerate) nanofibers	Cartilage defect	Mechanical support	[74]
Alginate	Excellent biocompatibility	Cryoelectrospinning of poly(ε‐caprolactone)	Cartilage defect	Anti‐inflammation and mechanical support	[259]
Chitosan and heparin	Rapid magnetic response	Super‐paramagnetic iron oxide nanoparticles	Cartilage defect	Vectoring delivery of biopharmaceuticals	[145]
Two‐component bioinstructive matrix (VitroGel‐RGD)	Ultrasound response	Barium titanate nanoparticles and GO nanoflakes	Cartilage defect	Piezoelectric, injectable, adheres to cartilage tissue, enhancing the mechanical strength, electrical conductivity, and bioactivity	[146]
Glycol chitosan/fucoidan	Good biocompatibility, injectability	Fu@KAFAK	OA	Anti‐inflammatory and regulation of ECM metabolic homeostasis	[163]
Gelatin	Good biocompatibility, injectability	Nanoliposomes‐loaded diclofenac sodium and kartogenin	OA	Self‐lubricating	[165]
Zein@alginate	Efficient delivery	Strontium@calcitriol	OA	Improving the hydration lubrication	[166]
HEMA‐methyl acrylamide (DMAA)	Soft elasticity and hydration characteristics, low friction	Liposomes	OA	Improving the lubrication and mechanical strength	[167]
Methacrylated hyaluronic acid	Efficient delivery	Polyhedral oligomeric silsesquioxane (POSS)	OA	Improving the lubrication and visualization	[258]
Gelatin	Efficient delivery, biocompatibility, and injectability	Polygallate–Mn nanoparticles	OA	Improving the lubrication and anti‐inflammation	[15c]
Poloxamer 407 (P407)/HA	Thermosensitive	Copper nanodots (Cu NDs)	OA	Anti‐inflammation	[171]
HA‐ADH	Self‐healing and lubrication	BSA–MnO	OA	Anti‐inflammation and pH responsive	[174]
Hyaluronic acid modified with aldehyde and methacrylic anhydride (AHAMA)	pH sensitive	Carbon quantum dots (CQDs)	OA	Anti‐inflammation and inhibiting and SB remodeling	[172]
GelMA	Excellent injectability and uniform size	KGN/Dex‐TSPBA	OA	Anti‐inflammation	[173]
N‐isopropyl acrylamide (NIPAM)	pH sensitive	Geraniol	OA	Anti‐inflammation	[260]
PVA–APBA–SF	Efficient delivery, injectability, and ROS sensitive	MSCs microvesicle (MV)	OA	Anti‐inflammation	[120a]
poly(N‐vinylcaprolactam)	Temperature‐responsive drug delivery	Poly(N‐vinylcaprolactam)	OA	Anti‐inflammation	[122b]
Mineralized hydrogel	In situ mineralization	Calcium phosphate nanocrystals	OA	Repair defects	[123]
poly‐L‐lactic acid (PLLA)	Counterbalance the overall polarization	PLLA nanofiber	OA	Electroinducing cartilage regeneration	[147]
Col II/HA/PEG	Good biocompatible	FeCl_3_	OA		[124b]
GelMA	Good biocompatible	T‐NNPs	IVDD	Anti‐inflammation	[205]
Alginate/gelatin	Bioink, mechanical support	Curcumin‐encapsulated polylactic acid nanoparticles	IVDD	Anti‐inflammation	[206]
Decellularized annulus fibrosus matrix (DAF) hydrogel	Good biocompatible	Polycarbonate nanoparticles‐loaded TrkA‐IN‐1	IVDD	Anti‐inflammation, analgesic effect	[207]
GelMA	Good biocompatible	Mineralized TGF‐β and CAT nanoparticles	IVDD	Anti‐inflammation, anti‐aging	[208]
Oxidized hyaluronic acid (OHA)/borax–gelatin	Injectable, self‐healing	PBNPs	IVDD	Scavenging ROS	[209]
GelMA	Injectable, mechanical support	BPQDs–chitosan	IVDD	Scavenging ROS, ameliorating extracellular acidosis	[210]
Chitosan–HA	Injectable	HAp‐epigallocatechin‐3‐gallate (EGCG) nanorods	IVDD	Inducing macrophage polarization	[212]
Gelatin methacrylate/hyaluronic acid methacrylate	Good biocompatibility and mechanical properties	C‐MSN‐T	IVDD	Inducing macrophage polarization, anti‐inflammation	[213]
SA/PNIPAAm	Good biocompatible, injectable	Silicate ceramics	IVDD	Inducing macrophage polarization	[215]
Gelatin	Good biocompatible, mechanical support	Gelatin nanospheres	IVDD	Inducing stem cell differentiation	[228]
Dextran/gelatin	Control released	PLGA‐loaded TGF‐β3	IVDD	Inducing stem cell differentiation	[229]
Glucose‐enriched decellularized nucleus pulposus hydrogel	Good biocompatible	LOX–MnO_2_ nanozyme	IVDD	Inducing stem cell differentiation	[231]
RAD/SA1, RADA16‐I (Ac‐RADARADARADARADA16‐1) and RSA1(AC‐(RADA)4‐GG‐EDVDHVFLRF‐CONH2)	Acid resisting	Self‐assembling nanofiber	IVDD	Inducing stem cell differentiation	[261]
Alginate	Excellent hydrophilicity	Ring‐aligned electrospun nanofibers	IVDD	Mechanical support	[197]
Chitosan‐poly(hydroxybutyrate*‐co*‐valerate)	Excellent hydrophilicity, viscoelastic	Chitosan–chondroitin sulfate nanoparticles	IVDD	Excellent complex shear modulus and stress relaxation values	[198]
FEFKFEFK (β‐sheet forming self‐assembling peptide) hydrogels	Mimicking the natural extracellular matrix	GO	IVDD	Mimicking the mechanical properties of the nucleus pulposus tissue	[201]
Chitosan/1‐ethyl‐3‐(3‐dimethylaminopropyl) carbodiimide hydrochloride (EDC)/N‐hydroxysulfo‐succinimide (NHS)	Surface adhesion	Calcium sulfate	IVDD	Calcium sulfate	[202]
Hyaluronic acid	Mimicking the natural extracellular matrix	Collagen and GAGs co‐precipitated	IVDD	Mimicking the mechanical properties of the nucleus pulposus tissue	[203]
CMC–GMA	Sensitive pH responsiveness	Tannic‐acid‐loaded antagomir‐21 nanoparticles	IVDD	ROS scavenging, anti‐inflammatory, and regulation of ECM metabolic homeostasis	[223]
Aldehyde‐functionalized hyaluronic acid (HA‐CHO) and poly(amidoamine) PAMAM	Novel injectable, self‐healing	siSTING@HP	IVDD	Anti‐inflammatory	[262]
Methacrylated hyaluronic acid (HAMA) microspheres	Good degradability, swellability, and injectability	Grafting circSTC2 silencing genes‐loaded 1,2‐dioleoyl‐3‐trimethylammonium‐propane/cholesterol/1,2‐dioleoyl‐sn‐glycero‐3‐phosphoethanolamine (DOTAP/Chol/DOPE) cationic liposomes	IVDD	Anti‐inflammatory and regulation of ECM metabolic homeostasis	[263]
OG/GCA	Fast gelation, injectability, and self‐healing	G5‐PBA	IVDD	Anti‐inflammatory and regulation of ECM metabolic homeostasis	[264]
Polyamine‐based PEG–polyplex nanomicelles	Efficient delivery	PEG–polyplex nanomicelles	IVDD	Regulation of ECM metabolic homeostasis	[265]
PEG‐b‐PDEAEM polymersomes	Dual stimuli sensitive (pH and NIR light)	Gold nanorods and doxorubicin (DOX)	IVDD	Encapsulation efficiency and accelerated DOX release under lower pH and NIR laser irradiation	[221]
PLGA–PEG–PLGA copolymer	Thermosensitive, capable of encapsulating MR409 and demonstrating ROS‐responsive release	mPEG20‐b‐PPS30 (PPS‐PEG)‐based vesicles loaded with MR409	IVDD	Inhibition of secretory autophagy and IL‐1β secretion, attenuation of oxidative stress‐induced effects, and promotion of extracellular matrix (ECM) homeostasis	[225]

#### 2.2.1 Polymer Matrix

The polymer matrix, the backbone of nanocomposite hydrogels, forms a 3D network that absorbs and retains water or biological fluids.^[^ ^31^ ^]^ Polymers can be natural (e.g., collagen,^[^ ^32^ ^]^ gelatin,^[^ ^33^ ^]^ alginate,^[^ ^34^ ^]^ hyaluronic acid [HA],^[^ ^35^ ^]^ silk,^[^ ^36^ ^]^ and chitosan^[^ ^37^ ^]^) or synthetic (e.g., poly(ethylene glycol) [PEG]),^[^ ^38^ ^]^ poly(vinyl alcohol) (PVA),^[^ ^39^ ^]^ poly(acrylic acid) [PAA],^[^ ^40^ ^]^ and polycaprolactone [PCL]^[^ ^41^ ^]^), each with unique advantages and disadvantages. Natural polymers offer biocompatibility and bioactivity but may have variable properties,^[^ ^31^ ^]^ while synthetic polymers provide greater control over properties but may lack inherent bioactivity.^[^ ^42^ ^]^ Recently, hybrid polymer matrices combining natural and synthetic polymers have gained interest for their enhanced properties and performance in tissue regeneration.^[^ ^43^ ^]^

#### 2.2.2 Nanomaterials

Nanomaterials are materials with dimensions between 1 and 100 nm on at least a 1D scale.^[7a]^ They can be ceramic (e.g., hydroxyapatite [HAP],^[^ ^44^ ^]^ bioactive glass,^[^ ^45^ ^]^ silica^[^ ^46^ ^]^), metallic (e.g., gold,^[^ ^47^ ^]^ silver,^[^ ^48^ ^]^ iron oxide^[^ ^49^ ^]^), polymeric (e.g., poly(lactic*‐co*‐glycolic acid) [PLGA],^[^ ^50^ ^]^ dendrimers^[^ ^51^ ^]^), or carbon‐based (e.g., carbon nanotubes (CNTs),^[^ ^52^ ^]^ graphene oxide (GO),^[^ ^53^ ^]^ nanodiamonds^[^ ^54^ ^]^).

Due to the special physicochemical properties of nanomaterials, their incorporation into hydrogels can significantly improve the performance of hydrogels and is expected to be used in various clinical applications. First of all, the small size of nanomaterials is the key to their entry into the cell interior to play a role, which can greatly improve the efficiency of drug action inside the cell. Nanomaterials enter cells by direct penetration, phagocytosis, cytotoxicity, receptor‐mediated endocytosis, and cytotoxicity.^[^ ^55^ ^]^ The selection of nanomaterials of appropriate sizes and their rational surface modification can significantly improve the efficiency of nanomaterials in cells.^[^ ^56^ ^]^ In addition, as the particle size of nanomaterials decreases, their surface area relative to volume increases rapidly, and the high surface‐to‐volume ratio (S/V ratio) that this brings has a significant impact on NP applications.^[^ ^57^ ^]^ Nanomaterials with high S/V ratios often have unique mechanical properties. Nanofibers and nanotubes, in particular, exhibit high strength and toughness, which can enhance the mechanical properties of composite materials.^[^ ^58^ ^]^ In addition, the high S/V ratios provide more chemical modification sites for attachment and modification of more bioactive molecules. On the one hand, this increases the efficiency of drug delivery and on the other hand enriches the functionality of nanomaterials. For example, Chen et al. modified mitochondria‐targeting peptides on the surface of metal polyphenol NPs via Schiff base reaction, enabling them to target mitochondria and repair mitochondrial function after entering nucleus pulposus cells.^[^ ^59^ ^]^ Currently, collagen II‐targeting peptides are also widely used in the modification of nanomaterials to actively target collagen II in cartilage tissue to achieve deeper access of nanomaterials into cartilage tissue.^[^ ^60^ ^]^ In addition, the morphology and surface chemistry of the nanomaterials had significant effects on the cells. Chen et al. altered the morphology and surface chemistry of controlled nanomaterials to investigate the interaction of the nanomaterial surface with macrophages and the osteogenic role of bone marrow mesenchymal stem cells (MSCs). They found that tuning surface chemistry and nanomorphology (16, 38, and 68 nm) modulated macrophage polarization and promoted osteogenic differentiation of MSCs.^[^ ^61^ ^]^

## 3 Fabrication Strategy

**Figure 1:**
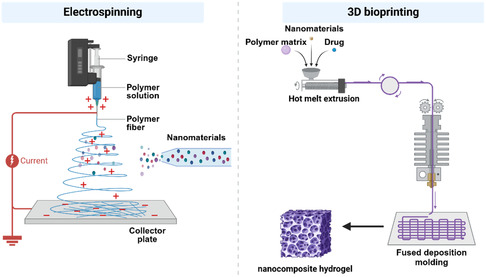
Schematic flow of electrostatic spinning and 3D bioprinting of nanocomposite hydrogels. Created with BioRender.com.

**Figure 2:**
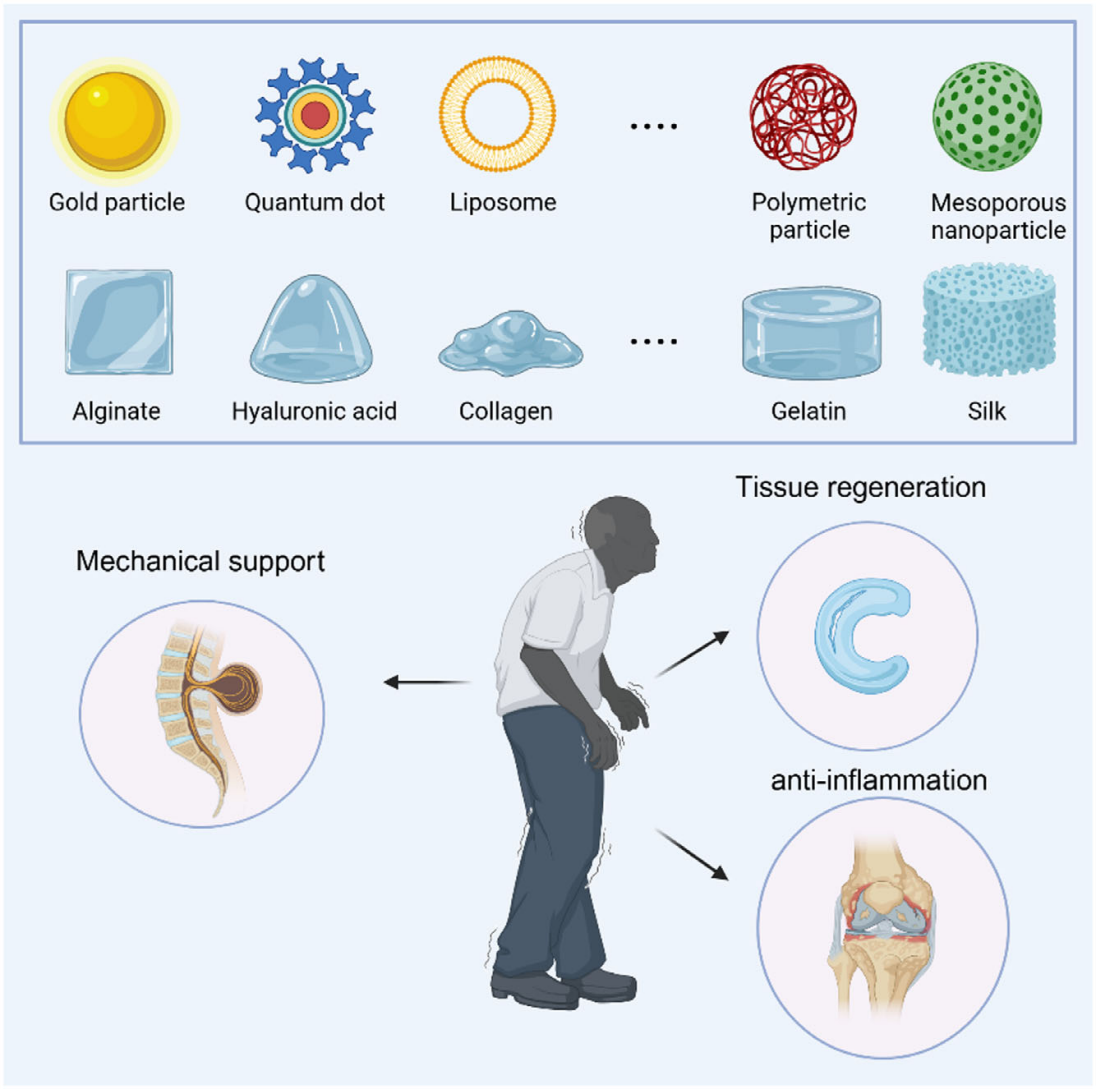
Different nanomaterials are combined with various polymer matrices to form nanocomposite hydrogels that provide mechanical support, tissue regeneration, and anti‐inflammatory functions needed for degenerative joint diseases. Created with BioRender.com.

In the previous section, we discussed the individual components of nanomaterials and hydrogels in nanocomposite hydrogels. In the following section, we will delve into the integration of nanomaterials with hydrogels to create nanocomposite hydrogels with enhanced functionalities. Nanomaterials play a pivotal role in enhancing the characteristics of nanocomposite hydrogels, with the fabrication process being key to their physicochemical properties (**Figure** **1**). The principal methods for fabricating these hydrogels—direct injection, electrospinning, and 3D printing—are selected based on application‐specific needs.^[^ ^62^ ^]^ In the synthesis of hydrogel, the timing of nanomaterial addition defines two different preparation methods: one involves incorporating nanomaterials before the gelation of the hydrogel, while the other entails adding nanomaterials post–pre‐gelation of the hydrogel (**Figure** **2**). The first category includes methods where NPs are integrated with the hydrogel precursors before cross‐linking, such as direct injection and 3D printing. This approach embeds NPs within the forming gel network.^[^ ^63^ ^]^ The second category features methods like electrospinning, where NPs are incorporated into a preformed hydrogel matrix post‐cross‐linking. This strategy allows for distinct property customization and application adaptability.^[^ ^64^ ^]^ Understanding the intricate relationship between fabrication techniques and the resulting properties of nanocomposite hydrogels is essential for their effective application across various domains. Each method offers unique advantages, enabling the fine‐tuning of hydrogel characteristics to fulfill specific requirements.

### 3.1 3D Bioprinting Nanocomposite Hydrogels

The 3D bioprinting of nanocomposite hydrogel scaffolds represents a cutting‐edge approach to cartilage repair, aiming to mimic the complex, layered structure of natural cartilage tissue. This technology enables precise control over the spatial distribution of cells, biomolecules, and NPs within the scaffold, allowing for the creation of structures that closely resemble native tissue. Several common 3D bioprinting technologies are used in bone tissue engineering, including extrusion‐based printing, inkjet printing, and laser‐based printing.^[^ ^65^ ^]^ These technologies allow for the layer‐by‐layer deposition of biomaterials to create complex 3D structures. In the context of nanocomposite hydrogel scaffolds, 3D bioprinting offers the ability to incorporate NPs into the hydrogel matrix, enhancing its mechanical properties and enabling controlled release of growth factors.^[^ ^66^ ^]^

Preparation of nanocomposite hydrogel scaffolds using 3D bioprinting involves the use of specialized bio‐inks that contain both the hydrogel precursor and NPs. A study by Odent et al. found that surface‐functionalized sulfonated silica NPs (SiO_2_–SO_3_–Na^+^) were employed as nanomaterials and incorporated into a hydrogel solution of pre‐polymerized acrylamide, [2‐(acryloyloxy)ethyl] trimethylammonium chloride, and N, N′‐methylenebisacrylamide. This composite material exhibits tunable hardness, resilience, ductility, and elasticity, enabling the rapid 3D printing fabrication of intricate architectures. Gelatin methacryloyl (GelMA) and HA methacrylate (HAMA) are also commonly used as bioinks for 3D bioprinting of nanocomposite hydrogel scaffolds.^[^ ^67^ ^]^ A study by Abbadessa et al. explores the use of triblock copolymer‐based hydrogels for cartilage bioprinting. Specifically, hydrogels based on PEG and partially methacrylated poly[N‐(2‐hydroxypropyl) methacrylamide mono/dilactate] (M10P10) support cartilage formation by chondrocytes. The incorporation of methacrylated chondroitin sulfate or HAMA improves the mechanical properties, long‐term stability, and printability of the hydrogels, highlighting their potential for cartilage repair strategies.^[^ ^68^ ^]^

The structural design of scaffolds in 3D bioprinting is a critical aspect of cartilage repair. By controlling the architecture and porosity of the scaffold, researchers can create an environment that promotes cell adhesion, proliferation, and differentiation.^[^ ^69^ ^]^ Additionally, the incorporation of NPs into the scaffold can improve its mechanical properties, ensuring that it can withstand the mechanical forces experienced in the joint.^[^ ^70^ ^]^ The influence of 3D bioprinting condition parameters, such as printing speed, nozzle size, and printing resolution, on the properties of the printed materials is an active area of research. Optimizing these parameters is essential for achieving the desired mechanical and biological properties of the scaffold.^[^ ^71^ ^]^

### 3.2 Electrospinning Nanocomposite Hydrogels

Electrospinning is an innovative technique that has gained significant attention in the field of material science and biomedical engineering due to its ability to produce 1D nanoscale fibers from a variety of polymer solutions. This method involves the use of a high‐voltage power supply, a metal needle syringe, and a grounding device to create an electric field that facilitates the ejection of a polymer jet, forming fine fibers as the solvent evaporates.^[^ ^72^ ^]^ The simplicity of the electrospinning process, coupled with its versatility in handling a wide range of natural and synthetic polymers, makes it an attractive option for fabricating nanofibers with tailored properties for diverse applications. One particularly promising area of application for electrospun nanofibers is in tissue engineering, where their high surface‐to‐volume ratio and porosity make them ideal candidates for scaffold materials.^[^ ^73^ ^]^ Further advancements in the field have led to the development of 3D porous nanofiber‐reinforced hydrogel scaffolds that closely mimic the ECM of cartilage. Gunes et al. achieved this by producing poly(3‐hydroxybutyrate*‐co*‐3‐hydroxyvalerate) nanofibers reinforced with carboxymethyl chitosan–silk fibroin hydrogel through wet electrospinning. The resulting composite scaffold maintained a stable interconnected microporous structure, which supported the differentiation of bone marrow stem cells.^[^ ^74^ ^]^ Hejazi et al. took this a step further by fabricating 3D nanofibrous scaffolds that closely resemble the native cartilage structure. The team hypothesized that by creating a gradient nanofiber scaffold composed of five layers, each with materials tailored to unique tissue structures such as PCL, gelatin, nanohydroxyapatite (nHAP), and chitosan, they could enhance mechanical properties and promote cell proliferation. This multilayered scaffold structure holds great potential for the treatment of degenerative joint diseases.^[^ ^75^ ^]^

## 4 Unique Properties of Nanocomposite Hydrogels

The diversity of nanomaterials and polymer matrices, along with the personalized preparation processes of nanocomposite hydrogels, endows nanocomposite hydrogels with unique properties, making them attractive candidates for applications in bone tissue engineering. These properties include the provision of mechanical properties, biocompatibility, biodegradability, injectability, antibacterial properties, and smart‐response release.

### 4.1 Mechanical Strength

Nanocomposite hydrogels offer superior mechanical strength, making them ideal for bone tissue engineering.^[^ ^76^ ^]^ NPs or nanofibers in the polymer matrix enhance the hydrogel's properties by consuming energy during stretching, which blunts crack tips and increases toughness and strength.^[^ ^77^ ^]^ Common nanofillers include carbon‐based NPs,^[^ ^78^ ^]^ metal and metal oxide NPs,^[^ ^79^ ^]^ polymer NPs,^[^ ^80^ ^]^ calcium‐phosphate‐based NPs,^[^ ^81^ ^]^ mesoporous silica NPs,^[^ ^82^ ^]^ mesoporous bioactive glass NPs,^[^ ^76^ ^]^ and clay NPs.^[^ ^83^ ^]^ Gao et al. developed an injectable nanocomposite hydrogel, chitosan NPs (CS‐NPs)@oxidized sodium alginate (OSA)‐l‐Gtn, with self‐healing and high mechanical strength. It combines CS‐NPs with dynamic polymer networks of OSA and gelatin (Gtn) using borax. The combined use of dynamic covalent bonds (like imine and borate ester bonds) and noncovalent interactions (such as electrostatic and hydrogen bonds) enhances energy dissipation, leading to high fatigue resistance and endurance under cyclic loading.^[^ ^84^ ^]^ Similarly, Aghajanzadeh et al. created Gel‐AXG (AXG—aldehyde‐modified xanthan gum [XG]) hydrogel with porous silicon NPs, which formed highly porous 3D microstructures with connected pores exhibiting enhanced mechanical properties, biomineralization, sustained release of silica ions, and excellent cytocompatibility with MG‐63 cells.^[^ ^85^ ^]^

In cartilage tissue engineering, embedding stiff nanomaterials within soft polymers significantly enhances mechanical properties. Piluso et al. used starch nanocrystals in gelatin hydrogels to increase compressive modulus and cell viability.^[^ ^86^ ^]^ nHAP in chitosan/collagen hydrogels, produced via the freeze‐gelation method, creates nanocomposite hydrogels with an interconnected porous structure and Young's modulus akin to native articular cartilage, promoting cell proliferation and differentiation.^[^ ^87^ ^]^ Additionally, polydopamine (PDA) NPs in dopamine‐modified alginate (Alg‐DA) hydrogels, cross‐linked with calcium ions, significantly improve mechanical properties.^[^ ^88^ ^]^ Additive manufacturing, particularly 3D printing, provides innovative methods for fabricating customized structures for cartilage tissue engineering. Sultan et al. utilized cellulose nanocrystals in sodium alginate (SA) and gelatin solutions to create ink materials, forming hydrogel scaffolds with uniform and gradient pore structures with improved mechanical properties.^[^ ^89^ ^]^ Incorporating GO into photo‐cross‐linkable alginate‐based hydrogels significantly enhances their mechanical properties by improving viscosity recovery and shape fidelity during 3D printing, leading to better scaffold resolution and cell proliferation for cartilage tissue engineering.^[^ ^90^ ^]^

Nanofibers and nanorods, with excellent mechanical properties and high aspect ratios, enhance hydrogels through physical or chemical doping.^[^ ^91^ ^]^ The study by Niu et al. developed polyacrylamide‐based nanocomposite hydrogels reinforced with cellulose nanofibers (CNF) and ferric ions, which significantly enhanced mechanical properties through dual physical cross‐links. The increased CNF and Fe3+ concentrations improved stiffness, toughness, and healing abilities, offering a promising approach for designing high‐strength, healable hydrogels.^[^ ^92^ ^]^

### 4.2 Biocompatibility

Nanocomposite hydrogels exhibit excellent biocompatibility, making them suitable for various biomedical applications, including bone tissue engineering. The biocompatibility of nanocomposite hydrogels is largely dependent on the choice of polymer matrix and nanomaterials used in their fabrication.^[^ ^93^ ^]^ Natural polymers, such as collagen, gelatin, and HA, are inherently biocompatible and can promote cell adhesion, proliferation, and differentiation.^[^ ^94^ ^]^ Synthetic polymers, such as PEG and PVA, are also biocompatible and have been widely used in the development of nanocomposite hydrogels.^[^ ^42^ ^]^ The biocompatibility of nanomaterials used in nanocomposite hydrogels is also crucial. The incorporation of nanomaterials can increase the mechanical strength and elasticity of nanocomposite hydrogels, making their mechanical properties closer to those of natural tissues, thereby reducing irritation and damage to surrounding tissues.^[^ ^95^ ^]^ Additionally, nanomaterials such as nanocellulose and nHAP can provide more cell adhesion sites, promoting cell adhesion and proliferation, which aids in tissue regeneration and repair.^[^ ^96^ ^]^ Moreover, nanomaterials can mimic the nanostructure of natural ECM, enhancing the biocompatibility of hydrogels, and allowing cells to grow and function better on them.^[^ ^97^ ^]^ However, the biocompatibility of some nanomaterials, such as CNTs and GO, remains a concern due to their potential cytotoxicity and immunogenicity.^[^ ^98^ ^]^ Therefore, the concentration and surface functionalization of these nanomaterials must be carefully optimized to ensure their biocompatibility in nanocomposite hydrogels.

### 4.3 Biodegradability

Biodegradability is another important property of nanocomposite hydrogels for bone tissue engineering applications. The ideal hydrogel should decompose at a stable rate, continuously releasing beneficial drugs. Its excellent biodegradability enables it to gradually adapt to the injured area during joint healing, reducing mechanical load, providing appropriate support, and ultimately being replaced by new tissue. Furthermore, the use of biodegradable materials can reduce or eliminate the need for secondary surgeries to remove implants. These materials naturally degrade within the body, significantly reducing the surgical risks and medical burdens for patients. The incorporation of NPs or nanofibers into the polymer matrix can influence the biodegradability of nanocomposite hydrogels.^[^ ^99^ ^]^ For example, a study by Pradhan et al. developed a biodegradable nanocomposite hydrogel based on chitosan and 2‐hydroxyethyl methacrylate (HEMA), with Kaolin added to enhance mechanical strength and biodegradability. By comparing the degradation rates of nanocomposite hydrogels with different Kaolin contents, it can be demonstrated that the incorporation of Kaolin NPs accelerates the degradation of the hydrogel. This acceleration is attributed to the increased hydrophilicity and porosity of the nanocomposite hydrogel due to the addition of Kaolin NPs. The enhanced hydrophilicity and porosity facilitate the penetration of water and other degradable components into the hydrogel matrix, thereby accelerating the degradation process of the nanocomposite hydrogel.^[^ ^100^ ^]^

### 4.4 Antibacterial Properties

Nanocomposite hydrogels significantly enhance their antibacterial capabilities by integrating different antibacterial components and mechanisms. During the preparation of nanocomposite hydrogels, the selection of nanomaterials, the choice of cross‐linking agents, the addition of antibacterial agents, the structural design of hydrogels, photothermal effects, electrical stimulation, biocompatibility and degradability, and environmental responsiveness significantly affect their antibacterial performance. For instance, nanomaterials such as silver NPs (AgNPs)^[^ ^101^ ^]^ and GO^[^ ^102^ ^]^ are widely used in the preparation of hydrogels due to their excellent antibacterial properties. Cross‐linking agents like N, N′‐methylene bisacrylamide (MBA) can enhance the mechanical properties and antibacterial properties of hydrogels.^[^ ^103^ ^]^ Antimicrobial agents such as metronidazole^[^ ^104^ ^]^ and curcumin magnesium polyphenol network (Cur–Mg)^[^ ^105^ ^]^ enhance antibacterial effects by releasing effective components. The design of dual‐network structures and Janus structures in hydrogels can significantly improve their antibacterial properties.^[^ ^104^ ^]^ Photothermal effects and electrical stimulation can also enhance the antibacterial effects of hydrogels to promote wound healing.^[^ ^106^ ^]^ Biocompatibility and degradability are key to ensuring that hydrogels degrade safely in the body and release antibacterial components.^[^ ^107^ ^]^ Environmentally responsive hydrogels can control the release of drugs by responding to external stimuli, thereby enhancing antibacterial effects. Balancing the antibacterial performance and biocompatibility of nanocomposite hydrogels is a key challenge in their preparation. Selecting appropriate nanomaterials,^[^ ^108^ ^]^ optimizing the concentration of nanomaterials, using biocompatible base materials, utilizing self‐assembly technology,^[^ ^109^ ^]^ combining electrical stimulation and antimicrobial peptides,^[^ ^110^ ^]^ designing environmentally responsive hydrogels, considering the surface modification of nanomaterials,^[^ ^108^ ^]^ and conducting in vitro and in vivo, evaluations are key strategies to achieve the best balance between antibacterial performance and biocompatibility of hydrogels.

### 4.5 Injectability

Injectability is a desirable property of nanocomposite hydrogels for bone tissue engineering, as it allows for minimally invasive delivery of the scaffold to the defect site.^[^ ^111^ ^]^ Injectable nanocomposite hydrogels can be easily molded to fit the shape of the defect, ensuring proper filling and integration with the surrounding tissue.

The injectability of nanocomposite hydrogels can be achieved by designing them to undergo a sol–gel transition in response to external stimuli, such as temperature, pH, or light.^[^ ^112^ ^]^ For example, Aghdam et al. developed an injectable chitosan hydrogel for bone tissue engineering by incorporating chitosan‐modified halloysite nanotubes loaded with icariin. This enhanced the hydrogel's mechanical strength and osteoinductivity. Notably, through sol–gel transformation, the hydrogel showed improved gelation characteristics, alongside biocompatibility, promoting cell proliferation and bone differentiation.^[^ ^113^ ^]^ Another approach to achieve injectability is the use of shear‐thinning nanocomposite hydrogels, which exhibit a decrease in viscosity under shear stress and rapidly recover their mechanical properties upon the removal of the stress.^[^ ^114^ ^]^ Liu and Yao created an injectable hydrogel using XG and methylcellulose (MC) with shear‐thinning properties. The blend solution's viscosity was reduced when subjected to shear stress at room temperature due to the XG network. At body temperature, the solution gelled as a result of the thermo‐responsive MC network. The hydrogel's properties could be adjusted by varying XG and/or MC concentrations. It showed biocompatibility and biodegradability in vivo, making it a promising material for long‐term drug delivery.^[^ ^115^ ^]^

### 4.6 Smart‐Response Release

In the progressive degeneration of the injured joint, the joint's microenvironment undergoes gradual changes.^[^ ^116^ ^]^ Effective drug release, specifically tailored to be highly specific and responsive to these changes, can help delay and halt the degenerative process of the joint.^[^ ^117^ ^]^ Nanocomposite hydrogels can be designed to respond to various external stimuli, such as temperature, pH, light, magnetic fields, electrical signals, or various internal responses, such as pH, ROS, and enzymes (**Table** **2**).^[^ ^118^ ^]^ These smart‐response releases allow for the dynamic control of the hydrogel's properties, enabling the development of smart scaffolds for bone tissue engineering.

**Table 2:** Stimuli‐responsive nanocomposite hydrogels for cartilage defect, OA, and IVDD treatment.

Category	Name	Main components	Characteristics	Research results	References
Cartilage defects	Magnetic‐response nanocomposite hydrogels	Chitosan, heparin‐based nanocomposite hydrogels, SPIONs, polyvinyl alcohol, nanohydroxyapatite, Fe_2_O_3_ nanoparticles, type‐II collagen, hyaluronic acid, polyethylene glycol, magnetic nanoparticles	Remote manipulation, rapid magnetic response, promotes chondrocyte activity, enhances mechanical properties and cell compatibility, stimulates chondrocyte‐related gene expression, responds to external magnetic fields while maintaining structural integrity	Promotes chondrocyte activity, enhances hydrogel performance, shows impressive mechanical properties and cell compatibility, stimulates expression of chondrocyte‐related genes, potential for cartilage regeneration	[124, 145, 266]
Cartilage Defects	Piezoelectric nanocomposite hydrogels	Barium titanate nanoparticles, GO nanoflakes, poly‐L‐lactic acid nanofibers, collagen	Boosts chondrogenic cell commitment in vitro, produces localized electrical cues under ultrasound activation	Enhances chondrogenic cell commitment, drives cartilage healing	[146, 147]
Cartilage defects	Photo‐responsive nanocomposite hydrogels	Various polymers, near‐infrared light, photopolymerized hydrogels	Precise, noninvasive control, fills cracks within cartilage, mimics native mechanical properties of cartilage, forms gel at body temperatures, photo‐cross‐linkable, mineralizable	Stabilizes injured cartilage, prevents post‐traumatic OA	[118, 123, 148, 149]
OA	Enzyme‐responsive nanocomposite hydrogels	Enzyme‐sensitive segments, RMTQ peptide, small molecules like TG‐18	Targets overactive enzymes like MMPs and ADAMTS, releases therapeutic agents upon enzyme contact, multiple capabilities like improved internal circulation stability, enhanced tissue penetration, site‐specific release	Provides better relief from arthritis symptoms, MMP‐12‐responsive drug release, promising for targeted OA treatment	[121, 177, 179]
OA	Temperature‐responsive nanocomposite hydrogels	Poly (N‐isopropyl acrylamide) (PNIPAM), poly (Ninylisobutyramide) (PAMAM), poly (2‐oxazoline) (POxs), poly [2‐(2‐methoxyethoxy) ethylmethacrylate] (PMEOMA), poly(N‐vinylcaprolactam) (νPVCL)	Sol‐gel transition at physiological temperatures, easy injection and in situ gelation, stable at normal body temperature, drug release at warmer temperatures	High loading capacity, sustained drug permeation, potential for topical drug delivery for OA pain management	[122, 180]
IVDD	pH‐responsive nanocomposite hydrogels	Polyanionic polymers, Ag@MSNs–PAA nanoparticles, polyethylene glycol‐block‐poly(N,N‐diethylaminoethyl methacrylate) (PEG‐b‐PDEAEM), gold nanorods, CA cross‐linked with PVA and AgNPs, CMC–GMA	Changes volume and structure in response to pH, controlled drug release in acidic environment, biocompatibility, degradability, tunable gelling time and mechanical strength, inhibition of MAPK/ERK signaling, reduction of TNF‐α expression	Reduces inflammation, promotes tissue regeneration, promising for IVDD repair	[219, 221, 222, 223]
IVDD	Inflammation‐response nanocomposite hydrogels	ROS‐responsive vesicles composed of PPS‐PEG amphiphilic polymers, thermosensitive PLGA‐PEG‐PLGA hydrogel, GLRX3+ extracellular vesicles (EVs‐GLRX3) hydrogels, nanohybrid peptide hydrogel (NHPH)	Tailored to respond to inflammatory microenvironment, drug release in response to ROS, modulates redox homeostasis, inhibits immune responses, restores regenerative microenvironments	Attenuates mitochondrial damage, mitigates nucleus pulposus senescence, restores ECM deposition, effective for structural and functional recovery of IVDs	[11, 225]

#### 4.6.1 Internal Responses

pH‐responsive hydrogels can be tailored to release bioactive agents or cells in response to local pH changes, with potential applications in IVD and cartilage replacement.^[^ ^119^ ^]^ ROS‐responsive hydrogels are designed to address inflammation and tissue damage in OA by releasing bioactive molecules in the presence of ROS.^[^ ^120^ ^]^ Enzyme‐responsive hydrogels target‐specific enzymes, such as matrix metalloproteinases (MMPs), involved in arthritis progression, allowing for targeted drug or cell delivery to affected joints.^[^ ^111^, ^121^ ^]^

#### 4.6.2 External Responses

Temperature‐responsive hydrogels transition from a sol to a gel state at physiological temperatures, facilitating easy injection and in situ gelation for defect repair, with potential use in OA pain management.^[^ ^122^ ^]^ Photosensitive hydrogels offer spatiotemporal control over their properties through cross‐linking or degradation upon light exposure, useful for treating acute mechanical damage to cartilage and preventing post‐traumatic OA.^[^ ^118^, ^123^ ^]^ Magnetically responsive hydrogels can be manipulated by external magnetic fields for remote property control or cell guidance, with promising applications in cartilage tissue engineering.^[^ ^124^ ^]^

## 5 Applications of Nanocomposite Hydrogels in Degenerative Joint Diseases

**Figure 3:**
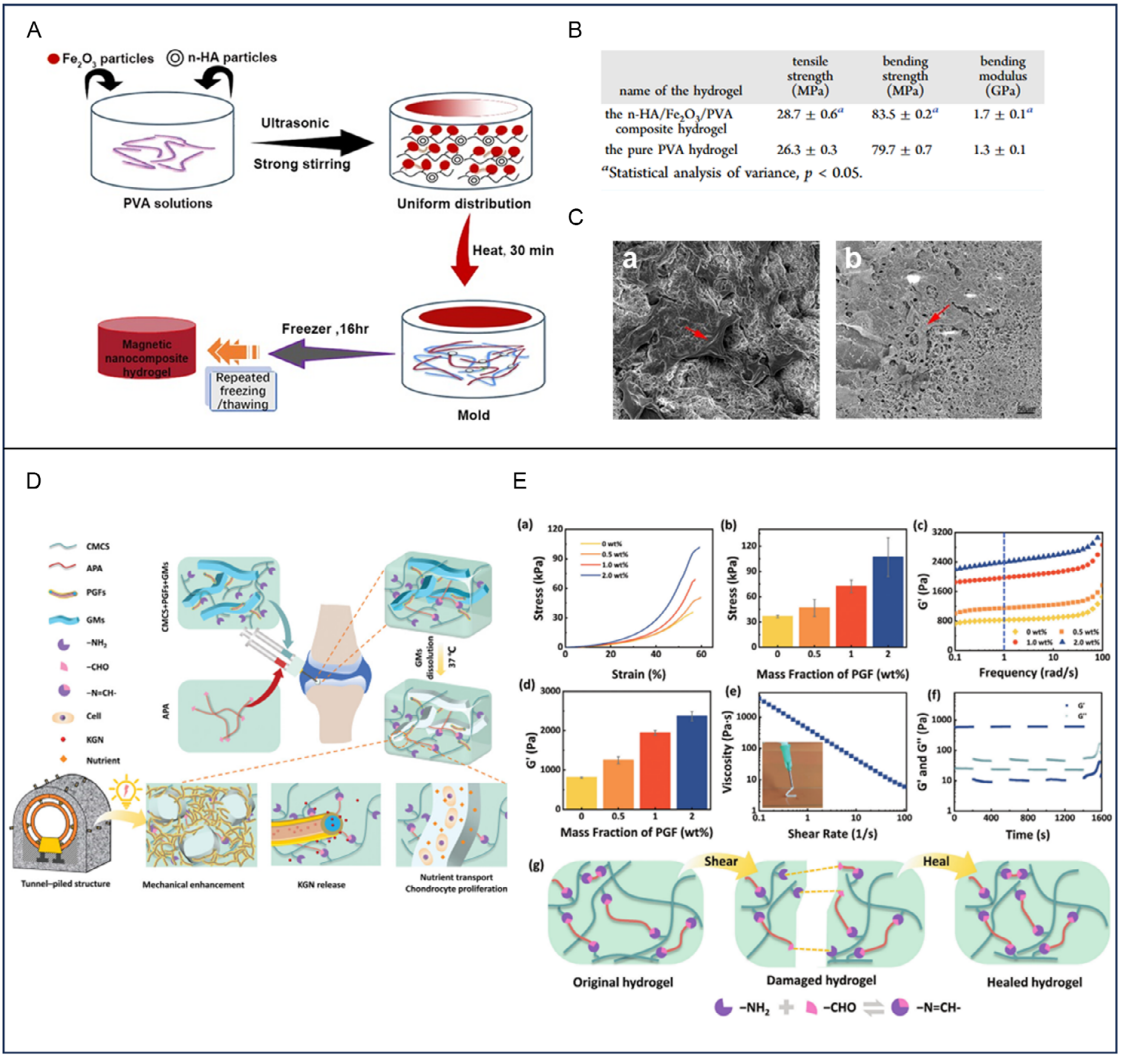
A) General scheme of the fabrication of n‐HA/Fe_2_O_3_/PVA hybrid magnetic nanocomposite hydrogel. B) Mechanical Properties of the n‐HA/Fe_2_O_3_/PVA composite hydrogel and the pure PVA hydrogel ($\bar{x}$ ± s, *n* = 10). C) Hydrogel material was cocultured with BMSCs for 1 week. a) A large number of spindle or polygonal cells adhered to and aggregated in the pores on the surface of the n‐HA/Fe_2_O_3_/PVA composite hydrogel. b) A small number of cells adhered to the pores on the surface of the pure PVA hydrogel. Reproduced (Adapted) with permission.^[130a]^ Copyright 2018, American Chemical Society. D) Schematic illustration of the preparation of nanofiber composite microchannel‐containing injectable hydrogels for cartilage tissue regeneration. E) a) Compressive stress–strain curves and b) the maximum compressive stress of nanofiber composite microchannel‐containing hydrogels with various PGF contents. c) Frequency dependence of *G*′ and d) *G*′ at 1 rad s^−1^ for the nanofiber composite microchannel‐containing hydrogels with various PGF contents. e) Viscosity–shear rate curve of the nanofiber composite microchannel‐containing hydrogels (3 wt% GMs, 2 wt% PGFs). f) G′ and G″ of nanofiber composite microchannel‐containing hydrogels in alternate step strain tests (3 wt% GMs, 2 wt% PGFs, high strain: 200%, low strain: 1%, interval time: 200 s). g) Schematic of the self‐healing mechanism of hydrogels. Reproduced (Adapted) with permission.^[^ ^131^ ^]^ Copyright 2023, John Wiley and Sons.

Common degenerative joint diseases include cartilage defects, OA, and IVDD. These conditions exhibit similar pathological changes during degeneration, primarily characterized by the loss and functional decline of resident cells within the joint. However, due to the distinct anatomical structures of different joint types, personalized treatment approaches are required to address their specific physiological and mechanical needs. Nanocomposite hydrogels represent a promising potential treatment method. By selecting appropriate nanomaterials and integrating them with hydrogels, novel nanocomposite hydrogels can be developed to meet diverse needs such as anti‐inflammatory effects, mechanical properties, tissue regeneration, environmental responsiveness, etc. (**Figure** **3**). Next, we will discuss the pathological characteristics of various degenerative joint diseases, current treatment limitations, and the application prospects of nanocomposite hydrogels.

## 6 Applications of Nanocomposite Hydrogels in Cartilage Defects

### 6.1 Pathophysiology of Cartilage Defects

Cartilage defects can arise from various factors, including trauma and age‐related degeneration. Trauma‐induced cartilage injuries, such as those caused by sports accidents or falls, can result in focal chondral or osteochondral lesions.^[^ ^125^ ^]^ These injuries can disrupt the complex structure and function of articular cartilage, leading to pain, swelling, and reduced joint mobility. Age‐related cartilage degeneration, in contrast, is characterized by the gradual degradation of the cartilage matrix, reduced chondrocyte viability, and altered mechanical properties.^[^ ^126^ ^]^ This degenerative process can be exacerbated by factors such as genetics, obesity, and joint overuse.

### 6.2 Limitations of Current Treatment Options

Current treatment options for cartilage defects, such as microfracture, autologous chondrocyte implantation (ACI), and osteochondral grafts, have several limitations. Microfracture involves creating small holes in the subchondral bone to stimulate the migration of bone‐marrow‐derived stem cells into the defect site.^[^ ^127^ ^]^ However, this technique often results in the formation of fibrocartilage, which has inferior mechanical properties compared to native hyaline cartilage. ACI involves harvesting and expanding chondrocytes from a healthy region of the patient's cartilage, followed by their implantation into the defect site.^[^ ^128^ ^]^ While ACI can produce hyaline‐like cartilage, it is associated with donor‐site morbidity, limited cell availability, and high costs. Osteochondral grafts, which involve transplanting bone and cartilage plugs from non‐weight‐bearing areas of the joint or cadaveric donors, can provide immediate structural support.^[^ ^129^ ^]^ However, these grafts are limited by donor site availability, potential immune rejection, and imperfect integration with the surrounding tissue.

### 6.3 Nanocomposite Hydrogels for Cartilage Defects Treatment

#### 6.3.1 Nanocomposite Hydrogels with Available Mechanical Properties

Various NPs and nanofibers have been incorporated into nanocomposite hydrogels to improve their mechanical properties, bioactivity, and chondrogenic potential. n‐HAP, which are the main inorganic component of bone, have been used to enhance the mechanical strength and osteoconductivity of nanocomposite hydrogels.^[^ ^130^ ^]^ Huang et al. developed a magnetic nanocomposite hydrogel using PVA, n‐HAP, and magnetic NPs (Fe_2_O_3_) (Figure 3A). The addition of n‐HAP enhanced the mechanical properties of the hydrogel, which is characterized by an increased tensile strength of ≈28.7 MPa, which is the maximum stress the hydrogel can endure before breaking under tension. Additionally, the bending strength, which measures the hydrogel's resistance to fracture when bent, is significantly higher at 83.5 MPa. The hydrogel also demonstrates a greater stiffness, with a bending modulus of 1.7 GPa, indicating its ability to provide robust structural support (Figure 3B). Coculture with bone mesenchymal stem cells (BMSCs) demonstrated uniform cell growth, high proliferation rates, and stimulated chondrocyte‐related gene expression, suggesting potential applications in cartilage tissue engineering (Figure 3C).

GO nanosheets have been shown to enhance the mechanical strength, electrical conductivity, and bioactivity of nanocomposite hydrogels.^[^ ^90^ ^]^ The compressive modulus of the hydrogel with a higher concentration of GO (ACG/GO1) (ACG is a composite of alginate, chondroitin and gelatin) was ≈60% greater than that of the hydrogels without GO and with a low concentration of GO (ACG and ACG/GO0.1, respectively). This improvement in stiffness is attributed to the GO nanofiller forming bridges that reinforce the 3D structure of the hydrogel scaffolds. Rheological tests revealed that the inks containing GO exhibited lower viscosities, which is beneficial for the extrusion process during 3D printing. Remarkably, the viscosity recovery time was drastically reduced in GO‐containing inks; ACG/GO1 regained 97% of its initial viscosity in just 1 s, compared to 64 s for ACG without GO.^[^ ^90^ ^]^ In addition, nanofibers were also used in nanocomposite hydrogels. Oylum Colpankan Gunes et al. developed a novel 3D porous nanofiber‐reinforced hydrogel composite scaffold that mimics the hydrated composite structure of the cartilage ECM. The scaffold was produced by dispersing wet‐electrospun poly(3‐hydroxybutyrate*‐co*‐3‐hydroxyvalerate) nanofibers within a carboxymethyl chitosan (CMCht)‐silk fibroin (CMCht‐SF) hydrogel matrix, chemically cross‐linked by PEG diglycidyl ether.^[^ ^74^ ^]^ Similarly, based on the aldehyde PEG/carboxymethyl chitosan (APA/CMC) hydrogel, Ricotti et al. used thermosensitive gelatin microrods (GMs) as the pore forming agent, and the coaxial electrospinning poly (lactic acid)/gelatin fibers (PGFs) loaded with carotene as the reinforcement and drug delivery system to construct the injection hydrogel nanofiber composite microchannel hydrogels (APA/CMC)/Kartogenin (KGN)@PGF/GM hydrogel) (Figure 3D). In particular, PGFs significantly improve the mechanical properties of hydrogels by forming interlaced fiber networks. Through dynamic mechanical analysis, it was found that the maximum compressive stress of the nanofiber composite microchannel hydrogels increased from 36 to 107 kPa with increasing PGF content. In addition, the compressive modulus varied in the range of 11.6–13.7 kPa and was not significantly different at different PGF contents. It was found by rheological tests that hydrogels with different PGF contents exhibited frequency‐dependent viscoelastic behavior, whereas the storage modulus (*G*′) varied little in the frequency range of 0.1–100 rad s^−1^. Moreover, the addition of PGF can greatly improve the self‐healing and shear properties of nanofiber composite microchannel hydrogels (Figure 3E).^[^ ^131^ ^]^

#### 6.3.2 Nanocomposite Hydrogels for Stem Cell Chondrogenic Differentiations

Various stem cell sources, including MSCs, adipose‐derived stem cells (ADSCs), and induced pluripotent stem cells (iPSCs), have been explored for cartilage tissue engineering using nanocomposite hydrogels. MSCs are the most widely studied stem cell type due to their chondrogenic potential and easy isolation from bone marrow, adipose tissue, and synovial tissue.^[^ ^132^ ^]^ A study by Zhao et al. developed a HA and CNF nanocomposite hydrogel with enhanced mechanical properties for cartilage tissue engineering, which supported bone marrow MSC proliferation and chondrogenic differentiation, demonstrating significant repair in a full‐thickness cartilage defect model in rats.^[^ ^133^ ^]^ ADSCs have also shown promise for cartilage regeneration due to their abundance and chondrogenic potential.^[^ ^134^ ^]^ Moya et al. developed GO‐incorporated bioconjugated hydrogel nanocomposite inks for 3D‐printed scaffolds, enhancing printability, shape fidelity, and human adipose‐tissue‐derived MSC proliferation. The bioconjugated hydrogel matrix induced chondrogenic differentiation, making these inks promising for cartilage tissue engineering using 3D printing.^[^ ^90^ ^]^ iPSCs, derived from the reprogramming of adult somatic cells, offer an unlimited cell source for cartilage tissue engineering.^[^ ^135^ ^]^ He et al. developed a synthetic‐scaffold‐free cartilage graft using murine iPSCs and embryonic stem cells on a 3D alginate‐based micro‐cavity hydrogel platform. The cells underwent mesoderm and chondrogenic differentiation, followed by removal of the alginate phase, resulting in grafts composed of chondrocyte cells and cartilaginous ECM.^[^ ^136^ ^]^

#### 6.3.3 Nanocomposite Hydrogels for Delivery of Growth Factors and Biomolecules

Due to the high surface‐to‐volume ratio of nanomaterials, they can bind and adhere to more bioactive factors. Consequently, the incorporation of nanomaterials enhances the retention time of nanocomposite hydrogels and enables controlled drug release. Nanocomposite hydrogels can be designed to deliver growth factors and biomolecules that promote cartilage regeneration. Transforming growth factor‐β (TGF‐β) is a potent inducer of chondrogenesis and has been widely used in cartilage tissue engineering.^[^ ^137^ ^]^ Insulin‐like growth factor‐1 (IGF‐1) is another important growth factor for cartilage homeostasis and regeneration.^[^ ^138^ ^]^ Moinzadeh et al. studied the effects of adjusting TGF‐β1, region‐specific growth factors, matrix stiffness, and nanofiber arrangement in hydrogels on the chondrogenic differentiation of 3D human MSCs (hMSCs), targeting superficial articular cartilage layers, middle layers, and calcified areas. Region‐specific matrix stiffness dominates hMSC differentiation toward superficial and calcified zone phenotypes, whereas aligned nanofibers enhance Col II expression in the superficial zone. IGF‐1 and TGF‐β1 have a dominant effect on cartilage differentiation in the mid zone.^[^ ^139^ ^]^ Bone morphogenetic proteins (BMPs), particularly BMP‐2 and BMP‐7, have also been shown to enhance chondrogenesis and cartilage repair.^[^ ^140^ ^]^ Sathy et al. investigated the influence of α‐tricalcium phosphate (α‐TCP) NP delivery into MSCs using an amphipathic cell penetrating peptide RALA on osteogenesis and bone formation. RALA‐complexed α‐TCP NP delivery to MSCs increased the expression of bone BMP‐2, upregulated a range of key osteogenic genes such as Runx2, Osterix, and alkaline phosphatase, and it enhanced the mineralization of MSC‐laden alginate hydrogels.^[^ ^141^ ^]^ PCL nanocomposite hydrogel also showed significant effects in delivering growth factors and biomolecules. For example, integrated dual‐function double‐layer 3D‐printed scaffolds (ECM/PCL coated with ECM hydrogel as the upper layer and MgO@PDA/PCL as the bottom layer) have been studied in vitro. They stimulate the proliferation, chondrogenic, and osteogenic differentiation of human bone marrow MSCs, demonstrating excellent repair capabilities.^[^ ^142^ ^]^ Additionally, silk fibroin hydrogels combined with CS‐NPs and functionalized with TGF‐β1 and BMP‐2 enhance cartilage repair by promoting chondrogenesis through sustained release of growth factors. The TGF‐β1@CS/BMP‐2@SF composite demonstrated excellent biocompatibility, improved cell viability, and effectively alleviated cartilage defects in both in vitro and in vivo models.^[^ ^143^ ^]^

#### 6.3.4 Nanocomposite Hydrogel for Smart‐Responsive Release

**Figure 4:**
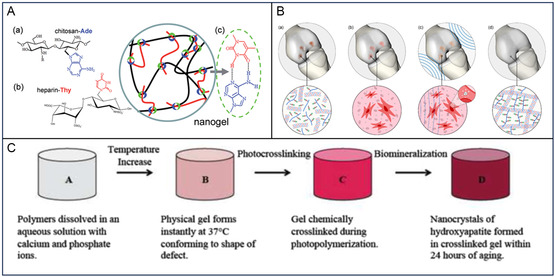
A) Schematic assembly of the nanocomposite hydrogel through the Watson–Crick nucleobase pairing between chitosan and heparin. The nanocomposite hydrogel precursors were designed with adenine and thymine on chitosan and heparin backbones, respectively, which are accessible for nucleobase pairing via hydrogen bonding. Reproduced (Adapted) with permission.^[^ ^145^ ^]^ Copyright 2014, The Royal Society of Chemistry. B) Depiction of the possible future therapeutic paradigm grounded on the hypothesis of this work. a) Degenerated cartilage tissue; b) application of the cell‐laden nanocomposite hydrogel in situ, c) stimulation with Ultrasound (US) waves, triggering the generation of intracellular local charges by exploiting nanomaterial piezoelectricity, and d) regenerated cartilage tissue. Reproduced (Adapted) with permission.^[^ ^146^ ^]^ Copyright 2024, American Chemical Society. C) Schematic depiction of the synthesis procedure for the fully mineralized hydrogel. It progresses to cartilage‐like material properties once it is fully mineralized. Reproduced (Adapted) with permission.^[^ ^123^ ^]^ Copyright 2011, Elsevier.

Magnetic‐response nanocomposite hydrogels are revolutionizing the field of cartilage tissue engineering with their unique ability to interact with external magnetic fields.^[124a]^ This interaction prevents the burst release of drugs, providing a prolonged and controllable drug release system that can be triggered and regulated by alternating magnetic fields.^[^ ^144^ ^]^ The study shows that magnetic bio‐nanocomposite hydrogels with specific nucleic acid base pairing can be used to deliver biological growth factors effectively. This magnetic nanocomposite hydrogel is constructed through Watson–Crick base pairing of chitosan and heparin via hydrogen bonding and is encapsulated in superparamagnetic iron oxide NPs to achieve a fast magnetic response. The presence of heparin allows for efficient adsorption of BMP‐2, and the release of BMP‐2 can be easily controlled by an external magnetic field. Under the control of the magnetic field, the viability of MG‐63 cells in this nanocomposite hydrogel is significantly enhanced (**Figure** **4**A).^[^ ^145^ ^]^ Further research has introduced a PVA‐based magnetic nanocomposite hydrogel, which, when combined with nHAP and magnetic NPs, demonstrates impressive mechanical properties and cell compatibility.^[130a]^ Notably, the inclusion of Fe_2_O_3_ NPs has been shown to markedly stimulate the expression of chondrocyte‐related genes, suggesting a promising scaffold material for cartilage tissue engineering. Additionally, a magnetic nanocomposite hydrogel containing type‐II collagen, HA, and PEG, along with magnetic NPs, has been found capable of responding to an external magnetic field for targeted actions while maintaining structural integrity,^[124b]^ further demonstrating its potential for cartilage regeneration. Magnetic nanocomposite hydrogels offer advantages such as controlled and timely drug release and the ability to influence chondrocyte behavior, providing new therapeutic approaches for treating degenerative joint diseases. Future research should focus on addressing the aggregation of magnetic materials within the hydrogels and improving their stability.

The use of piezoelectric nanomaterials combined with ultrasound stimulation is emerging as a promising approach for wirelessly triggering the regeneration of different tissue types. Ricotti et al. show that adipose‐tissue‐derived MSCs embedded in hydrogels containing piezoelectric barium titanate NPs and GO nanoflakes and stimulated with ultrasound waves with precisely controlled parameters (1 MHz and 250 mW cm^−2^, for 5 min once every 2 days for 10 days) dramatically boost chondrogenic cell commitment in vitro (Figure 4B).^[^ ^146^ ^]^ Vinikoor et al. present an injectable, biodegradable piezoelectric hydrogel, made of short electrospun poly‐L‐lactic acid nanofibers embedded inside a collagen matrix, which can be injected into the joints and self‐produce localized electrical cues under ultrasound activation to drive cartilage healing.^[^ ^147^ ^]^

Photo‐penetration technology offers precise, noninvasive control in designing biomaterials, leveraging light to adjust drug delivery systems’ behavior through intensity, location, and exposure control.^[^ ^148^ ^]^ This technology has led to the development of various photo‐responsive carriers, supporting applications from surface to deep tissue treatments, including using near‐infrared (NIR) light for cartilage defects.^[^ ^149^ ^]^ Schlichting et al. synthesized a photopolymerized nanocomposite hydrogel aimed at treating acute mechanical damage to cartilage (Figure 4C).^[^ ^123^ ^]^ This hydrogel is designed to fill in cracks within cartilage, simulating the native mechanical properties of the cartilage itself. It can form a gel at body temperatures, is capable of being photo‐cross‐linked in situ, and can mineralize, forming a nanocomposite that could potentially stabilize injured cartilage and prevent post‐traumatic OA.^[^ ^118^ ^]^

## 7 Applications of Nanocomposite Hydrogels in OA

### 7.1 Pathophysiology of OA

OA is a multifaceted disease extending beyond cartilage defects, encompassing synovial inflammation, subchondral bone changes, and other pathological alterations.^[^ ^150^ ^]^ The degeneration of articular cartilage is a hallmark of OA, resulting from an imbalance between anabolic and catabolic processes in chondrocytes.^[^ ^151^ ^]^ This imbalance leads to the degradation of the cartilage ECM, loss of chondrocytes, and impairment of the tissue's mechanical properties.^[^ ^152^ ^]^ Synovial inflammation, or synovitis, is another key feature of OA, characterized by the infiltration of inflammatory cells, production of pro‐inflammatory cytokines, and increased expression of matrix‐degrading enzymes.^[^ ^153^ ^]^ Synovitis contributes to the progression of OA by exacerbating cartilage degradation and causing pain and swelling in the affected joint.^[^ ^154^ ^]^ Subchondral bone remodeling, involving increased bone turnover, sclerosis, and the formation of osteophytes, is also a significant component of OA pathophysiology. The altered mechanical properties of the subchondral bone can further contribute to the degeneration of the overlying articular cartilage.^[^ ^155^ ^]^

### 7.2 Limitations of Current Treatment Options

Current treatment options for OA primarily focus on symptom management and pain relief rather than disease modification or cartilage regeneration. Nonsteroidal anti‐inflammatory drugs (NSAIDs) are commonly prescribed to reduce pain and inflammation in OA patients.^[^ ^156^ ^]^ However, long‐term use of NSAIDs can lead to adverse effects, such as gastrointestinal ulcers, cardiovascular events, and renal dysfunction.^[^ ^157^ ^]^ Intra‐articular drug injections are another treatment option for OA, providing short‐term pain relief and reduced inflammation.^[^ ^158^ ^]^ Nonetheless, the dynamic nature of joint fluid metabolism within the joint cavity complicates the control of drug concentration, leading to a brief residence time for the medication. This necessitates frequent injections to maintain therapeutic effectiveness.^[^ ^159^ ^]^ Additionally, repeated drug injections can cause cartilage damage, subchondral bone necrosis, and an increased risk of infection.

Nanocomposite hydrogels offer a promising solution to the limitations of current OA treatments. These hydrogels can be engineered to provide sustained release of therapeutic agents, such as anti‐inflammatory drugs or growth factors, directly to the affected joint, minimizing the need for frequent injections.^[^ ^160^ ^]^ Their tunable properties allow for controlled drug release, overcoming the challenge of maintaining therapeutic drug levels in the joint cavity.^[^ ^161^ ^]^ Furthermore, the incorporation of NPs, such as cerium oxide NPs with antioxidant properties, can help mitigate the adverse effects of long‐term NSAID use by reducing oxidative stress and inflammation in the joint.^[^ ^162^ ^]^ The mechanical properties of nanocomposite hydrogels can also be tailored to match those of native cartilage, providing mechanical support to the damaged joint and promoting tissue regeneration. Additionally, the use of nanocomposite hydrogels can reduce the risk of infection associated with repeated injections, as the hydrogel matrix can serve as a barrier to pathogens.^[^ ^163^ ^]^ Overall, nanocomposite hydrogels hold great promise as a disease‐modifying treatment for OA, offering targeted and sustained delivery of therapeutics while minimizing adverse effects and improving patient outcomes.

### 7.3 Nanocomposite Hydrogels for OA Treatment

#### 7.3.1 Nanocomposite Hydrogel Available for Intra‐Articular Injection

Nanocomposite hydrogels can be easily injected into the affected joint using minimally invasive techniques, such as arthroscopy or ultrasound‐guided injection.^[^ ^164^ ^]^ This minimally invasive delivery allows for the localized administration of the therapeutic agents directly to the site of cartilage degeneration, reducing systemic side effects. Additionally, the injectable nature of these hydrogels enables them to conform to the shape of the joint space, providing a scaffold for cell infiltration and tissue regeneration.^[15c]^ A study by Li et al. developed glycol chitosan/fucoidan nanocomposite hydrogels loaded with the anti‐inflammatory peptide (KAFAK) microfluidic hyaluronic acid methacrylate spheres (GC/Fu@KAFAK NGs) for intra‐articular injection to inhibit inflammation and attenuate OA progression. The nanocomposite hydrogels demonstrated anti‐inflammatory and chondro‐protective effects on interleukin‐1β (IL‐1β)‐stimulated rat chondrocytes, reducing the expression of inflammatory factors and enhancing chondrogenic markers. In a rat OA model, IA injection of GC/Fu@KAFAK NGs reduced glycosaminoglycan loss and inflammatory cytokine release, indicating their potential for OA treatment.^[^ ^163^ ^]^ A study by Chen et al. investigated the therapeutic effect of sinomenium (SIN) encapsulated by chitosan microspheres (CM–SIN) and photo‐cross‐linked GelMA hydrogel for OA. The combination promoted chondrocyte autophagy and ameliorated cartilage matrix degradation induced by IL‐1β in chondrocytes and an ex vivo model. In a surgically induced OA mouse model, intra‐articular injection of CM–SIN and GelMA hydrogel retarded OA progression by inducing autophagy.^[^ ^164^ ^]^

#### 7.3.2 Nanocomposite Hydrogels with Lubrication and Mechanical Support

OA is commonly recognized as a disease associated with inadequate joint lubrication. During the development of OA, joint friction gradually increases, which not only causes sustained physical damage to the joint surfaces at the physical level but also leads to the release of a large number of inflammatory factors, which reduce the activity of chondrocytes at the molecular level and lead to disturbances in the metabolism of the cartilage ECM. Therefore, reducing friction in OA is an effective means of treating this disease.

**Figure 5:**
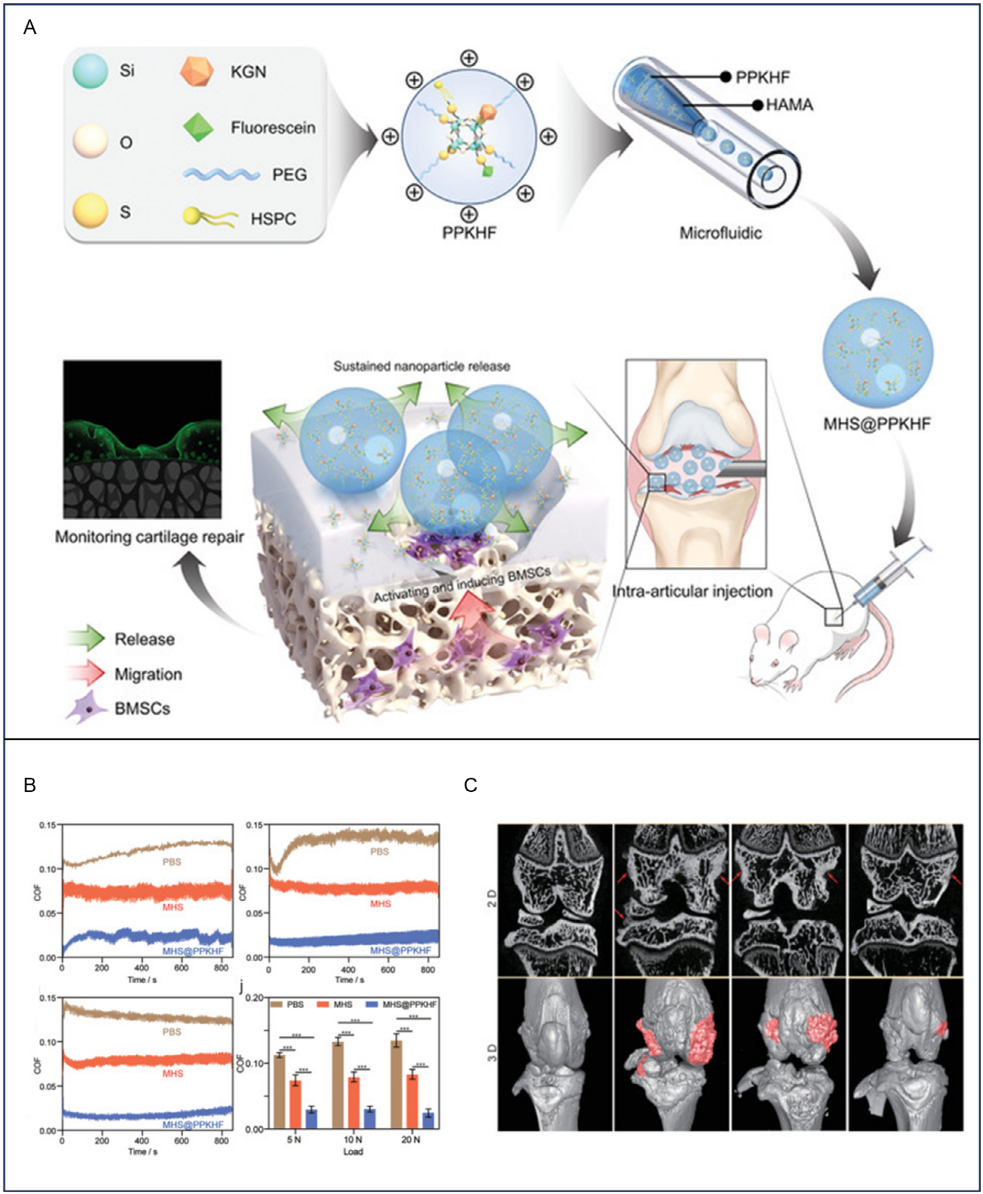
A) Based on the concept of “treatment‐monitoring integration,” a micro–nano double lubricating hydrogel microsphere with functions of in situ cartilage regeneration and repair process visualization was designed, which realized the monitoring of tissue regeneration process. B) Friction curves and statistical graphs and C) micro‐CT tomographic images and 3D reconstruction images of rat knee joints at 8 weeks postoperation. Reproduced (Adapted) with permission.^[^ ^258^ ^]^ Copyright 2023, John Wiley and Sons.

Liu et al. drew inspiration from the composition and function of menisci to develop an injectable nanoliposome‐encapsulated hydrogel with self‐lubricating and friction‐responsive characteristics. The hydrogel served as a carrier for the nanoliposomes loaded with anti‐inflammatory diclofenac and cartilage‐regenerating KGN, which exerted the anti‐inflammatory effect of diclofenac and cartilage‐regenerating effect of KGN while ensuring continuous exposure of the nanoliposomes to form a hydrated layer, thereby reducing friction during exercise.^[^ ^165^ ^]^ To address cartilage sliding interface lubrication dysfunction, Xie et al. developed superlubricative zein@alginate/strontium@calcitriol (ZASC) nanospheres to treat advanced OA. ZASC are nanospheres that can deliver medications and can reduce the amount of friction between joints. Calcitriol was encapsulated in zein, and subsequently, the zein@alginate was encapsulated by an alginate–Sr polymer to form a hydrated layer to reduce friction. Under the condition of simulated joint fluid, the friction coefficient of the ZASC + HA group was significantly lower than that of the HA group, which proved that ZASC had good lubricating properties and could effectively reduce the friction coefficient.^[^ ^166^ ^]^ The phospholipid molecule consists of two hydrophobic fatty acyl tails attached to a highly hydrated amphiphilic ionic phosphatidylcholine head capable of attracting water molecules to form a tough hydrated layer. Xiao et al. prepared a lipid hydrogel with high‐strength mechanomechanics and remarkable lubrication properties inspired by the mechanism of phospholipids in joint lubrication. In the presence of lipids, the lubrication ability of the lipid hydrogel was significantly improved with a low coefficient of friction (COF) of 0.026, which is about 5.3 times lower compared to the blank hydrogel.^[^ ^167^ ^]^ In addition, Yao et al. successfully prepared fluorescent nanomaterials for cartilage repair based on sulfhydryl polyhedral oligomeric silsesquioxane (POSS‐SH) by linking PEG, KGN, hydrogenated soybean phosphatidylcholine, and fluorescein using a “click chemistry” method. Subsequently, PPKHF NPs were encapsulated in microfluidic hyaluronic acid methacrylate spheres (MHS) to form MHS@PPKHF microspheres, which were injected in situ into the joint cavity using microfluidics (**Figure** **5**A). Due to the presence of phospholipid head groups with lubricating properties in the nanomaterial PPKHF, a lubricating film can be formed on the cartilage surface, enhancing the lubrication performance of the articular surface and reducing the physical wear of cartilage due to mechanical friction. Friction experiments indicate that the COFs of the PBS group are the highest under different loads, followed by the MHS group, with the lowest COF observed in the MHS@PPKHF group. Furthermore, the COF of the MHS@PPKHF group under a high load of 20 N did not only increase but also decreased to 0.024, fully meeting the requirements for joint lubrication (Figure 5B). In vivo experiments have also proved that it can reduce the osteophyte production of joints and maintain joint space height by reducing friction (Figure 5C). Considering that friction induces an inflammatory response within the joints, Chen et al. added polygallate–Mn (PGA–Mn) to the OSA/gelatin hydrogel (OGPGM), and the hydrogen bonding and electrostatic interactions between a large number of phenolic hydroxyl groups in PGA–Mn and water molecules led to excellent hydration and lubrication of the OGPGM hydrogel, and the polyphenols, as antioxidants, likewise exerted an excellent anti‐inflammatory effect that reduced the expression of inflammatory factors in the joints.^[15c]^

#### 7.3.3 Nanocomposite Hydrogels with Anti‐Inflammatory and Antioxidant Properties

What significantly distinguishes OA from the treatment of cartilage defects is that OA is a long term chronic persistent inflammatory arthritis, so research based on anti‐inflammatory as well as antioxidant properties is crucial.^[^ ^168^ ^]^ Current research suggests that chondrocytes subjected to biomechanical and biological factors in the early stages of OA generate a large amount of stress, causing chondrocytes to produce large amounts of ROS and inflammatory factors, which exacerbate the cross talk of inflammation and tissue damage and accelerate the progression of OA.^[^ ^169^ ^]^ Therefore, antioxidant and anti‐inflammatory biomaterials hold promise for the treatment of OA.

**Figure 6:**
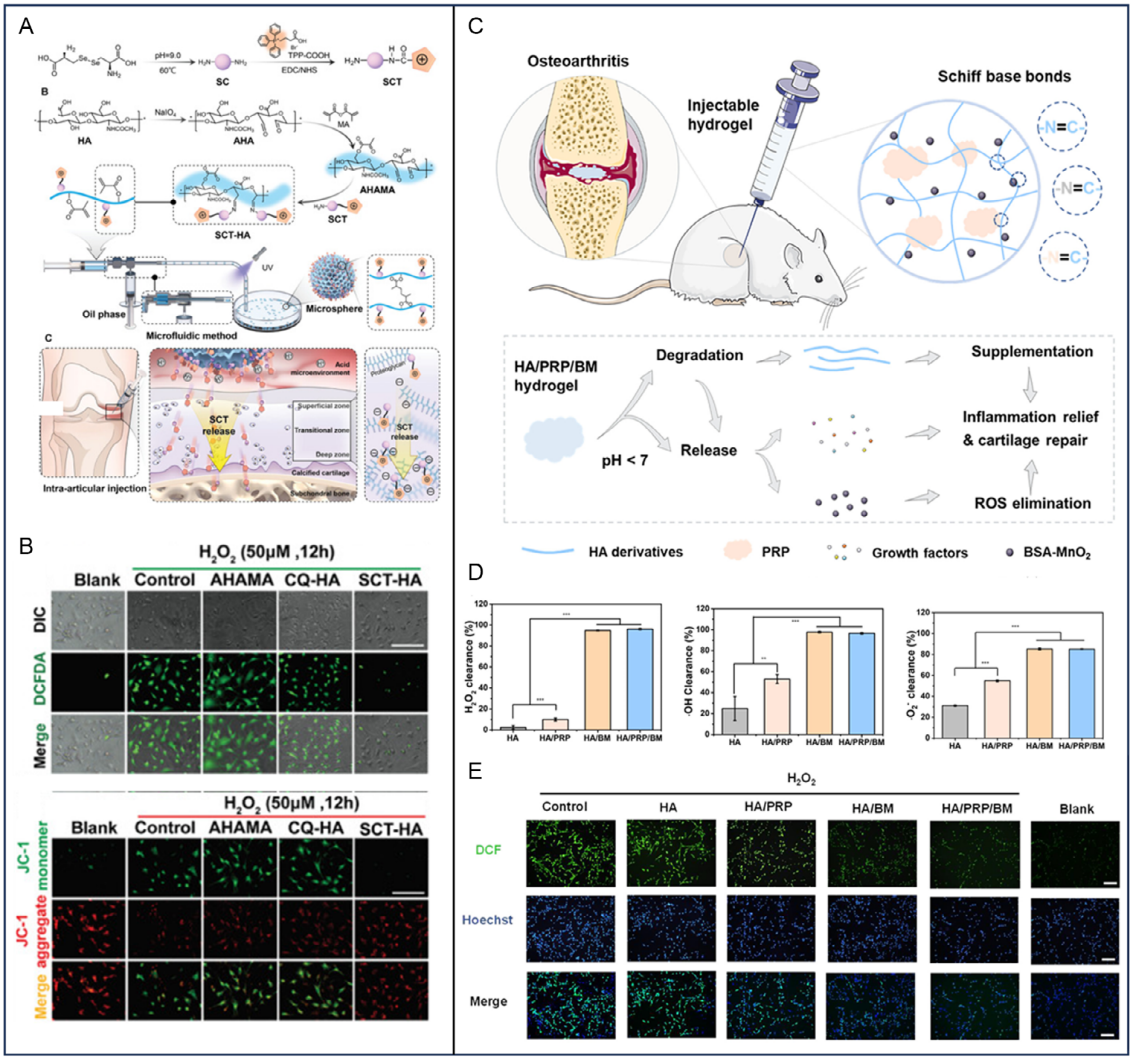
A) SCT synthesis, SCT‐HA preparation using microfluidic technology, which includes the synthesis process of AHAMA, and SCT‐HA release SCT continuously in a weakly acidic environment within the joint cavity, allowing it to penetrate the cartilage matrix and reach the subchondral bone (SB) for OA treatment. B) Inhibition of H_2_O_2_‐induced ROS production by micro/nanohydrogel microspheres, 2',7'‐Dichlorofluorescin diacetate (DCFH‐DA) staining of chondrocytes (green) indicating H_2_O_2_‐induced ROS production; scale bar = 100 μm. and J‐Aggregate Forming Chromophore (JC‐1) stain images of depolarized mitochondrial membranes after H_2_O_2_ intervention in chondrocytes. Scale bar = 100 μm. Reproduced (Adapted) with permission.^[^ ^172^ ^]^ Copyright 2024, John Wiley and Sons. C) Schematic illustration showing the injectable hydrogel of HA/PRP/BM fabricated via Schiff base reaction, and its synergetic treatment of OA owing to viscosupplementation, ROS elimination, inflammation relief, and cartilage repair promotion. D) Depletion test of hydrogels for H_2_O_2_, •OH, and •O_2_^−^. E) Intracellular ROS generation measured by DCF. Reproduced (Adapted) with permission.^[^ ^175^ ^]^ Copyright 2022, Elsevier.

The ultrasmall size of copper endows it with potent free radical scavenging ability, while also possessing the capacity to stimulate cartilage regeneration.^[^ ^170^ ^]^ Zhu et al. utilized a blend comprising HA and poloxamer 407 (P407) as a thermosensitive gel scaffold. They incorporated Cu NDs and platelet‐rich plasma (PRP) into the gel matrix to fabricate HA‐P407‐PRP (HPP)@Cu hydrogels. The HPP@Cu gel demonstrated efficient reactive oxidative nitrogen (RON) scavenging and reduced RON levels in the joint microenvironment.^[^ ^171^ ^]^ Zhou et al. used the antioxidative ability of selenium‐doped quantum dots to reduce oxidative stress in subchondral bone. They designed and synthesized highly permeable micro/nanohydrogel microspheres by using aldehyaluronic acid modified with methacrylate anhydride (AHAMA) and selenium‐doped carbon quantum dots grafted with triphenylphosphine (SCT) to extend the residence time of quantum dots in the joint cavity (SCT‐HA) (**Figure** **6**A). Experiments show that the SCT‐HA group containing quantum dots has the highest ROS clearance efficiency and the best repair ability for ROS damage (Figure 6B).^[^ ^172^ ^]^ Inspired by the phenomenon of bees tracking the color of flowers to phone nectar, Yu et al. developed an ROS‐responsive NP (KGN/Dexamethasone(Dex)‐N1‐(4‐boronobenzyl)‐N3‐(4‐boronobenzyl)‐N1,N1,N3,N3‐tetramethylpropane‐1,3‐diaminium (TSPBA) subchondral bone), then prepared injectable hydrogel microspheres (HMs), and finally immobilized the ROS‐responsive NPs with a type‐II collagenase‐targeting peptide in a GelMA hydrogel by microfluidic technology. This nanohydrogel system reduced inflammatory factors in chondrocytes by scavenging excess ROS within the chondrocytes thereby reducing inflammatory factors within the chondrocytes. Considering that MnO_2_ nanoenzymes have excellent antioxidant capacity and catalyze the production of Mn^2+^ from MnO_2_ in the weakly acidic environment of OA, this is an ion that can promote the differentiation of MSCs toward chondrocytes. Zhou et al. innovatively crafted nano‐enzymatic encapsulated hydrogels, incorporating bovine serum albumin (BSA)‐MnO_2_ (BM) NPs within an HA/PRP gel network, cross‐linked via Schiff base reaction (Figure 6C).^[^ ^174^ ^]^ Hybrid hydrogel doped with BM NPs demonstrated efficient scavenging of different kinds of ROS (Figure 6D) and could effectively remove ROS from chondrocytes and reduce oxidative stress in chondrocytes (Figure 6E), showcasing its promising therapeutic efficacy in OA management. In addition, nanofibrous membranes made of PCL and lignin copolymers were developed for OA treatment. Lignin has intrinsic antioxidant activity while PCL modulates mechanical properties. It was shown that PCL‐lignin nanofibers displayed excellent antioxidant and anti‐inflammatory effects in H_2_O_2_‐stimulated human chondrocytes and OA rabbit models. These nanofibers inhibited ROS production through an autophagic mechanism and activated antioxidant enzymes.^[^ ^175^ ^]^

#### 7.3.4 Smart‐Responsive Release Nanocomposite Hydrogels for OA Dynamic Microenvironment

Enzyme‐responsive nanocomposite hydrogels represent an innovative strategy for targeted OA treatment,^[^ ^111^ ^]^ capitalizing on the overactivity of specific enzymes like MMPs and a disintegrin and metalloproteinase with thrombospondin motifs (ADAMTS) that are instrumental in cartilage degradation and joint inflammation.^[^ ^176^ ^]^ These hydrogels are engineered to release therapeutic agents upon contact with these enzymes, ensuring that the medication is delivered directly to the affected sites. The nanocomposite hydrogels are composed of a nanomaterial scaffold with enzyme‐sensitive segments that disintegrate when they come into contact with the aberrantly expressed enzymes in the joint, thereby releasing the encapsulated therapeutic agents.^[^ ^177^ ^]^ This triggered release mechanism is designed to occur through various pathways, including size reduction, surface charge alteration, ligand activation, and chemical bond scission.^[^ ^178^ ^]^ The system can be endowed with multiple capabilities, such as improved internal circulation stability, enhanced deep tissue penetration, and site‐specific release, which collectively aim to increase drug bioavailability and minimize adverse side effects. The development of these hydrogels involves the synthesis of peptides and molecules that are sensitive to the enzymes present in the OA‐affected joints. For example, the RMTQ peptide (a potent anti‐inflammatory peptide derived from Annexin A1, enhanced with a cell‐penetrating peptide(T), a MMP‐2/9 diagestible peptide(M), and an inflammation‐targeting RGD peptide(R)) has been shown to respond effectively to MMP‐2/9, exhibiting stronger intracellular transport capacity and a higher rate of pro‐inflammatory factor degradation, thus providing better relief from arthritis symptoms in mouse models.^[^ ^179^ ^]^ Moreover, the integration of small molecules like triglycerol monostearate (TG‐18) into an NP‐hydrogel hybrid system has demonstrated the potential for MMP‐12‐responsive drug release, offering a controlled and on‐demand therapeutic approach.^[^ ^121^ ^]^ While these enzyme‐responsive hydrogel systems are still under development and face several technical challenges, they hold significant promise for the future of OA treatment. The precise targeting of enzymes involved in OA pathology allows for a more nuanced and effective treatment strategy, potentially transforming the management of this debilitating condition.

Temperature‐responsive nanocomposite hydrogels can be designed to undergo a sol–gel transition at physiological temperatures, allowing for easy injection and in situ gelation at the defect site.^[122a]^ Thermo‐responsive nanocarriers use heat from treatments like radiofrequency thermal ablation, microwave, or ultrasound to release drugs at naturally warmer disease sites. They're designed to be stable at normal body temperature but release drugs when slightly warmer. Key materials include poly (N‐isopropyl acrylamide) (PNIPAM), poly (Ninylisobutyramide) (PAMAM), poly (2‐oxazoline) (POxs), and poly [2‐(2‐methoxyethoxy) ethylmethacrylate] (PMEOMA),^[^ ^180^ ^]^ making them promising for arthritis treatment where affected areas are already warmer. For example, Zavgorodnya et al. engineered temperature‐responsive nanocomposite hydrogel multilayers of poly(N‐vinylcaprolactam) (νPVCL) for topical drug delivery, particularly targeting OA pain management. These hydrogel films demonstrated high loading capacity and sustained permeation of a nonsteroidal anti‐inflammatory drug, sodium diclofenac, through artificial skin membranes. This study highlights their potential as skin‐sensitive materials for delivering drugs relevant to OA treatment.^[122b]^

#### 7.3.5 Nanocomposite Hydrogels for Deep Cartilage Penetration in OA

The dense cartilage matrix in articular cartilage forms a physical barrier, while the heavily negatively charged proteoglycans within the collagen fiber network create an electrostatic barrier, both of which impede drug diffusion and transport.^[^ ^181^ ^]^ Positively charged nanomaterials of small size are crucial for overcoming these barriers and achieving effective drug penetration.^[^ ^181^, ^182^ ^]^ However, due to the continuous renewal of synovial fluid and the action of various enzymes and other proteins, the retention time of nanomaterials within the joint cavity is short, and some may become inactivated.^[^ ^183^ ^]^ Therefore, selecting an appropriate carrier is essential to ensure effective nanomaterial penetration. Nanocomposite hydrogel systems, by loading nanomaterials with unique physicochemical properties, can deeply penetrate cartilage tissue and ensure efficient, timely, and sustained release of the nanomaterials (**Table** **3**). Lin et al. constructed injectable adhesive HMs loaded with charge‐directed nanoscale secondary structures PDA@Lipo@HAMA. First, the synthesis of gallic acid (GA)‐conjugated boron‐glucan (PHB‐glucan) enabled ROS‐reactive drug release. Second, positively charged liposomes with PHB–glucan as the core were prepared. Finally, the liposomes were loaded on dopamine modified HAMA microspheres with HA and methacrylic anhydride as raw materials. Positively charged liposomes can penetrate the dense, negatively charged cartilage structure. These liposomes are encapsulated within HMs to prevent their rapid clearance by the capillaries and lymphatic system within the joints. In the OA model of rats, the injectable PDA@Lipo@HAMA microspheres effectively penetrated the cartilage matrix successfully delivered drugs to chondrocytes under oxidative stress and inhibited chondrocyte apoptosis.^[^ ^184^ ^]^ A study by Geiger et al. demonstrated that conjugating a growth factor to a cationic nanocarrier enhances delivery and efficacy in treating osteoarthritis. The nanocarrier, using reversible electrostatic interactions with anionic cartilage tissue, improves tissue binding, penetration, and residence time. Optimal PEGylated PAMAM dendrimers showed 70% uptake in cartilage and 100% cell viability. When conjugated to IGF‐1, the dendrimer penetrated cartilage within 2 days and extended IGF‐1 joint residence time by tenfold for up to 30 days. In a rat osteoarthritis model, a single dendrimer‐IGF‐1 injection reduced cartilage degeneration width by 60% and osteophyte burden by 80%, suggesting improved pharmacokinetics and efficacy of osteoarthritis drugs.^[^ ^185^ ^]^ Zuo et al. synthesized ultrasmall selenium‐doped carbon quantum dots using a hydrothermal method. These dots were dynamically complexed with AHAMA to construct highly permeable micro/nano‐HMs (SCT@AHAMA). Due to the ultrasmall 0D size (≈5 nm) of these quantum dots, they can easily penetrate the dense structure of cartilage. Furthermore, when combined with the AHAMA gel, their effective, rapid, and prolonged action within the joint is ensured.^[^ ^172^ ^]^ Given that the localization and accumulation of drugs within the joint determine their efficacy, Lin and colleagues designed an injectable doubly cationic HM with targeted ECM, cartilage penetration, and cellular phagocytosis capabilities. First, the surface of these HMs carries a positive charge, allowing them to adhere to the negatively charged cartilage surface. Additionally, the positively charged Kartogein loaded within these microspheres further penetrates the cartilage under the guidance of these charges. In a rat model of knee osteoarthritis, the targeted doubly cationic HMs effectively overcame the barriers to joint drug delivery, inhibiting cartilage matrix degradation and subchondral bone changes, demonstrating their potential as a therapeutic approach for osteoarthritis.^[^ ^186^ ^]^ Lei et al. developed injectable HMs with self‐renewable hydration layers to alleviate osteoarthritis. These HMs, made from HA and incorporating rapamycin‐loaded liposomes, enhance cartilage penetration. The liposomes, averaging 102.3 ± 35.2 nm in size, can penetrate the dense cartilage matrix due to their small size and positive charge. This facilitates interaction with negatively charged cartilage and ensures effective intracellular transport of rapamycin.^[^ ^187^ ^]^ Despite the positive findings, further research is necessary to ensure the safety and efficacy of these nanosystems before they can be applied in the clinic.

**Table 3:** Nanocomposite hydrogels for deep cartilage penetration in OA treatment.

Material design	Animal model	Therapeutic effects	Material functional advantages	Cartilage penetration advantages and source	References
PAMAM dendrimers (generations 4–6) PEGylation	Anterior cruciate ligament transection + partial medial meniscectomy (ACLT + MMx)	Dendrimer‐IGF‐1 conjugates showed better results than free IGF‐1, reducing cartilage degeneration and osteophytes	Sustained release of growth factors to chondrocytes with observed biocompatibility and no acute or chronic toxicity. PEGylation reduces dendrimer toxicity and improves biocompatibility, extending IGF‐1's intra‐articular half‐life to 30 days.	Small size and tunable surface charge enable deep cartilage penetration. Reversible electrostatic interactions with anionic proteoglycans and PEGylation provide steric hindrance for deeper tissue penetration.	[185]
Poly‐l‐lysine (PLL)‐PAMAM microspheres	ACLT + MMx	Microspheres enhanced drug delivery to the joint, reduced cartilage and subchondral bone degradation, and delayed OA progression.	Positively charged PLL targets cartilage, while PAMAM improves matrix penetration and cellular uptake. Sustained drug release of kartogenin (KGN) for over 21 days.	PAMAM's cationic nature allows it to cross the negatively charged cartilage ECM. The nanocarrier's small size penetrates the dense collagen network. Charge guidance helps microspheres overcome cartilage ECM barriers.	[186]
Rapamycin(RAPA)@Lipo@HMs	ACLT + MMx	RAPA@Lipo@HMs improved joint lubrication via smooth rolling and self‐renewable hydration layers.	Self‐renewable hydration layers sustain lubrication despite coating damage. Rapamycin from cationic liposomes targets cartilage and maintains cellular homeostasis.	Liposomes (<200 nm) easily penetrate cartilage. Positive zeta potential targets negatively charged glycosaminoglycan chains. Cationic liposomes are readily internalized by chondrocytes for RAPA delivery.	[184]
Dopamine‐modified hydrogel microspheres	Mono‐iodoacetic acid (MIA)‐induced OA model	Injectable microspheres deliver antioxidants to chondrocytes in OA. Reduced chondrocyte apoptosis under oxidative stress.	Overcomes the dense structure and high‐density negative charge of the cartilage matrix. ROS‐responsive release improves drug utilization and efficacy.	Positively charged liposomes aid hydrogel microspheres in penetrating the cartilage matrix. Enables deep penetration and controlled drug release in the cartilage extracellular matrix.	[172]
Se‐CQDs with AHAMA microspheres	ACLT	Inhibits osteoclastogenesis and H‐vessel invasion. Prevents cartilage degeneration in subchondral bone.	Selenium in SCT scavenges ROS, inhibiting osteoclast differentiation and chondrocyte apoptosis. SCT‐HA microspheres release SCT in weakly acidic environments, penetrating cartilage and reaching subchondral bone.	The ultrasmall size (≈5 nm) and positive charge of SCT enhance its ability to penetrate the cartilage matrix. Hydrogel microspheres allow controlled delivery and effective cartilage interaction.	[187]

## 8 Applications of Nanocomposite Hydrogels in IVDD

### 8.1 Pathophysiology of IVDD

The IVDs are elastic joints that connect the upper and lower vertebrae. They comprise a central nucleus pulposus, outer annulus fibrosus (AF), and cartilaginous endplates.^[^ ^188^ ^]^ The nucleus pulposus contains type‐II collagen and proteoglycan, forming a porous network that enhances elasticity, acting as a shock absorber during movement. The AF, made of type‐I collagen fibers, surrounds the nucleus pulposus, allowing rotation and bending while preventing nucleus pulposus displacement.^[^ ^189^ ^]^ The cartilaginous endplate facilitates nutrient transfer from the spinal capillaries to the disc.^[^ ^190^ ^]^ IVDD is a complex degenerative process mainly related to genetics, obesity, environment, and trauma.^[^ ^191^ ^]^ Normal nucleus pulposus tissue is constantly in a dynamic balance between nucleus pulposus cellular senescence, apoptosis, and oxidative and antioxidant downturns. However, in pathological conditions, this balance is disrupted and oxidative stress erupts, leading to the production of massive inflammation. Excessive inflammatory factors lead to premature senescence, macrophage M1 polarization, and activation of apoptotic pathways, resulting in a decrease in myeloid cell viability and number.^[^ ^192^ ^]^ Moreover, research has demonstrated that throughout the advancement of IVDD, collagen, and proteoglycans within the ECM undergo enzymatic cleavage, resulting in a decrease in the ECM's water content. This phenomenon is intricately linked to the heightened expression of MMPs after pathological alterations in myeloid cells.^[^ ^193^ ^]^ The breakdown of the ECM causes changes in the mechanical properties of the original IVD, leading to destabilization in the vertebral body and continued stress stimulation of the IVD during motion, which ultimately accelerates IVDD.^[^ ^59^ ^]^

### 8.2 Limitations of Current Treatment Options

Current treatment options for IVDD include conservative management and surgical interventions, both of which have limitations in terms of long‐term efficacy and potential complications. Conservative management typically involves physical therapy, pain medication, and lifestyle modifications.^[^ ^194^ ^]^ While these approaches can provide symptomatic relief, they do not address the underlying disc degeneration or promote regeneration of the damaged tissue. Surgical interventions, such as discectomy and spinal fusion, aim to remove the herniated disc material and stabilize the affected spinal segment.^[^ ^195^ ^]^ However, these procedures are associated with risks, such as infection, nerve injury, and adjacent segment degeneration, and may not always result in long‐term pain relief or functional improvement.^[^ ^196^ ^]^ The emergence of biomaterial‐based therapies, particularly nanocomposite hydrogels, offers a new horizon of hope. These innovative materials combine the robust biomechanical support of hydrogels with the precision of nanoscale components, enabling the controlled release of various bioactive substances. They are designed to penetrate the nucleus pulposus and interact with cellular pathways to promote the regeneration and repair of the degenerated disc, potentially reversing IVDD and restoring the spine's natural function.

### 8.3 Nanocomposite Hydrogels for IVDD Treatment

#### 8.3.1 Nanocomposite Hydrogels for Biomechanics and Biomimetic Tissue Engineering

**Figure 7:**
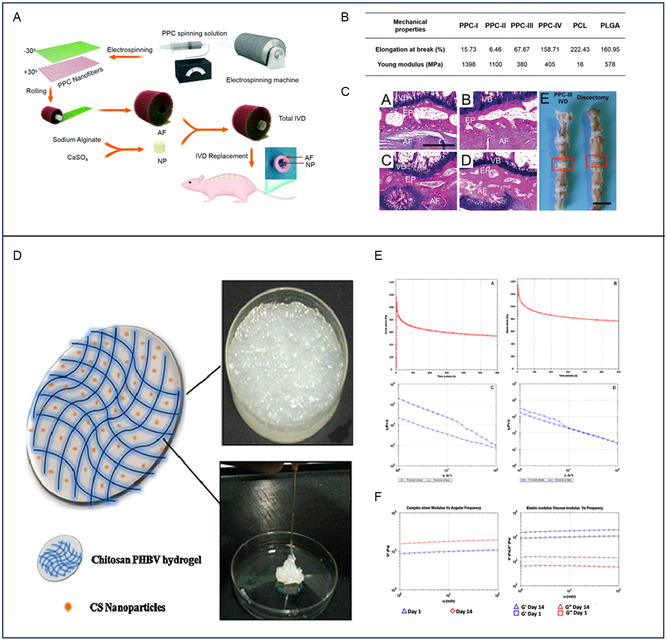
A) The schematic illustration of the procedure of IVD preparation and replacement. B) Elongation properties and Young's modulus of the electrospun(ES) sheets. C) The histological analysis of the tissue engineered (TE)‐IVDs. The hematoxylin and eosin (HE) staining shows the tissue interface of the native IVD. Reproduced (Adapted) with permission.^[^ ^197^ ^]^ Copyright 2017, The Royal Society of Chemistry. D) Physicochemical characterization of chitosan–poly(hydroxybutyate‐*co*‐valerate) (PHBV) hydrogel. E) Stress relaxation experiments. F) Biodegradation studies. Reproduced (Adapted) with permission.^[^ ^198^ ^]^ Copyright 2015, Elsevier.

The IVD is the joint that connects the upper and lower vertebrae and acts as a shock absorber during movement. Therefore, the selection of nanocomposite hydrogels with mechanical support in degraded IVDs is a priority. Yang et al. prepared hydrophilic PCL/PLGA/collagen (PPC) nanofiber sheets by blending a natural hydrophilic material, type‐I collagen, with a PCL/PLGA mixture by electrostatic spinning to increase the hydrophilicity and mechanical stability of the scaffolds and mimic the natural environment. This multilayer AF based on PPC nanofibers and NPs based on alginate hydrogel formed a fully functional artificial IVD (**Figure** **7**A). The incorporation of nanofibers further decreased the elongation capability of the PPC electrospun scaffolds and increased Young's modulus, effectively mimicking the unique mechanical properties of IVDs (Figure 7B). This provides a potential therapeutic approach for IVDD.^[^ ^197^ ^]^ Interestingly, after implantation of the PPC electrospun scaffolds, the scaffold fully integrates into the natural disc tissue interface and that disc joint can move like a normal disc joint (Figure 7C). This suggests that the PPC electrospun scaffolds can be used as a novel replaceable artificial disc.

Nair et al. developed a composite hydrogel for nucleus pulposus tissue engineering using chitosan‐poly(hydroxybutyrate*‐co*‐valerate) (CP) with chondroitin sulfate NPs without a cross‐linker (Figure 7D). Nucleus pulposus, as a viscoelastic tissue, experiences immediate significant relaxation upon strain, followed by gradual relaxation. In stress relaxation experiments, CP–chondroitin sulfate hydrogels with chondroitin sulfate addition exhibit higher relaxation stress values compared to CP hydrogels. Additionally, the nucleus pulposus behaves as a non‐Newtonian fluid, meaning it thins under shear (with viscosity decreasing overtime under constant shear). At lower shear rates, the viscosity of CP–chondroitin sulfate hydrogels is significantly higher than that of CP hydrogels (Figure 7E,F).^[^ ^198^ ^]^ Dewle et al. developed a PCL‐supported electrocompacted type‐I collagen patch (A‐PCL‐NH2 + Col‐I) for AF repair. The scaffold utilizes surface‐modified electrospun‐aligned PCL nanofibers, which provide high tensile strength, moduli, and water absorption. The PCL nanofibers are integrated with electro‐compacted type‐I collagen to create a biomechanically robust and hydrophilic scaffold.^[^ ^199^ ^]^ In addition, a study by Li et al. demonstrated that a nanofiber‐reinforced SA hydrogel with silk fibroin core–shell structures significantly enhanced nucleus pulposus regeneration. The silk fibroin nanofibers provided mechanical support and facilitated cell proliferation while enabling leak‐proof delivery of PRP, resulting in reduced degeneration in rat models.^[^ ^200^ ^]^

GO stands out due to its high water dispersibility and excellent biocompatibility. Ligorio et al. found that incorporating GO as a nanofiller can enhance the mechanical properties and hydration characteristics of hydrogels.^[^ ^201^ ^]^ In addition, the existing hydrogels have poor mechanical properties in resisting disc loading and lack adhesion to IVD tissue, resulting in displacement and extrusion of the hydrogel from the AF defect. Recent advances in bioadhesive hydrogels highlight their ability to adhere to biological tissues and tolerate biomechanical loads. Li et al. proposed that tissue‐mimicking bioadhesives can reproduce the biomechanical and cellular properties of the target tissue, thereby maximizing the repair effect. There may be further implications for disc repair in the future.^[^ ^202^ ^]^ Yang et al. chemically modified collagen and HA and co‐precipitated them with glucosamine polysaccharide to prepare a nanomaterial similar to nucleus pulposus tissue, namely aminated collagen‐aminated HA–glycosaminoglycan (aCol–aHA–GAG). Figure 7E demonstrates the disc height recovery of the biphasic IVD scaffolds after the mechanical test. The results indicated that nanomaterials containing GAG precipitate exhibited advantages in restoring IVD height.^[^ ^203^ ^]^

#### 8.3.2 Nanocomposite Hydrogels for Anti‐Inflammatory and Antioxidant Applications

**Figure 8:**
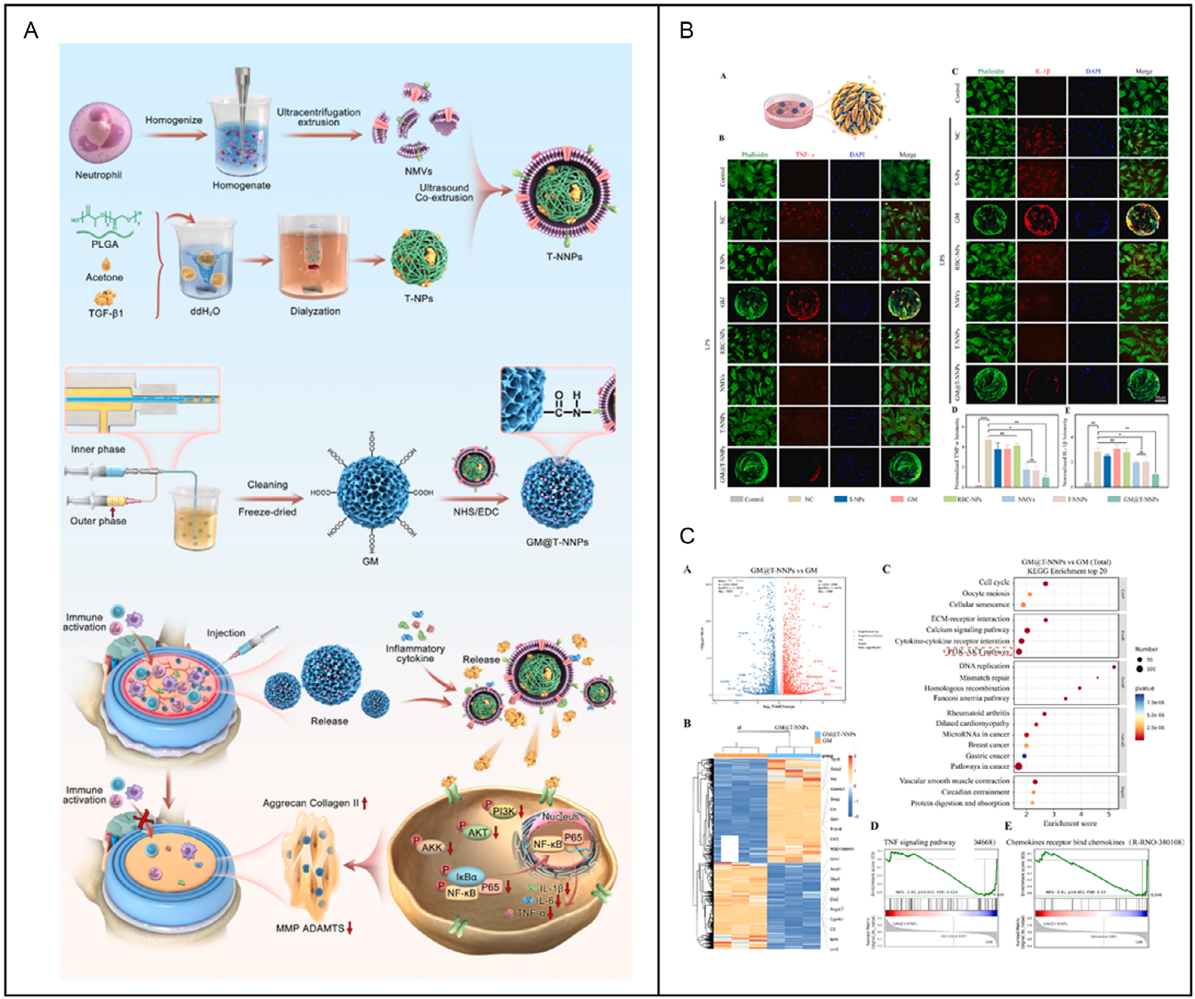
A) Design of immune‐defensive microsphere to block inflammatory cascade and promote regeneration of the nucleus pulposus. B) Inflammation control effect of GM@T‐NNPs C) Transcriptome sequencing analysis of the mechanism by which composite microspheres downregulate the inflammatory microenvironment of nucleus pulposus cells. Reproduced (Adapted) with permission.^[^ ^205^ ^]^ Copyright 2024, Elsevier.

Current research suggests that inflammation and oxidative stress play crucial roles in the process of IVDD. Therefore, targeting anti‐inflammatory and antioxidant interventions within the IVD represents a potential therapeutic approach for treating IVDD.^[^ ^204^ ^]^ The anti‐inflammatory effect of nanocomposite hydrogels is mainly dependent on the NPs or nanofibers contained in the hydrogel and other anti‐inflammatory drugs. Liang et al. injectable HMs functionalized with neutrophil membranes. TGF‐β1‐loaded PLGA NPs encapsulating neutrophil membranes (T‐NNPs) and GelMA were amide‐bonded to construct microsphere‐NP “inflammation camouflage” complexes (GM@T‐NNPs), and the NPs were uniformly distributed on the surface of the microsphere carriers (**Figure** **8**A). GM@T‐NNPs significantly inhibited LPS‐induced inflammation in nucleus pulposus cells in vitro, downregulated the expression of inflammatory factors and matrix MMPs, and upregulated the expression of collagen type II and aggrecan (Figure 8B).^[^ ^205^ ^]^ In addition, total RNA sequencing revealed phosphatidylinositol 3‐kinase (PI3K) and RAC‐alpha serine/threonine‐protein kinase associated with inflammatory cascade amplification under GM@T‐NNP treatment (Protein Kinase B (AKT)) related pathways were significantly inhibited, and it was verified by western blotting experiments that the therapeutic effect of GM@T‐NNP was related to the PI3K‐AKT signaling pathway (Figure 8C).

Similarly, Zamboni et al. developed curcumin‐encapsulated polylactic acid NPs for anti‐inflammatory purposes. These NPs, ranging from 50 to 250 nm, were incorporated into an alginate/gelatin hydrogel. The hydrogel, cross‐linked with calcium chloride, showed biocompatibility and suppressed tumor necrosis factor (TNF)‐α production in THP‐1 cells, suggesting the potential for tissue engineering to reduce inflammation.^[^ ^206^ ^]^ Peng et al. developed a composite hydrogel (TI–cNP–DAF) for treating disc‐related pain. The decellularized AF (DAF) matrix hydrogel, containing amphiphilic polycarbonate NPs loaded with a nerve growth factor receptor antagonist (TrkA‐inhibitor (IN)‐1), inhibited neuroinflammation and sensitization. TI–cNP–DAF showed potential for treating discogenic pain by mediating the inflammatory environment and inhibiting nerve ingrowth.^[^ ^207^ ^]^ Zheng et al. created hydrogen ion‐capturing HMs (minerialized nanoparticle‐loaded GelMA microsphere, GMNP) with mineralized TGF‐β and catalase (CAT) NPs to reverse inflammation. GMNP neutralizes acidic microenvironments, releases TGF‐β and CAT, and inhibits NLRP3 activation. In vitro, GMNP suppressed the TXNIP/NLRP3/IL‐1β axis and boosted ECM synthesis. In vivo, GMNP reduced inflammation and fostered IVD regeneration.^[^ ^208^ ^]^

Nanohybrid peptide hydrogel (NHPH) with 2D‐MnO_2_ nanosheets can provide ROS scavenging while producing oxygen as a by‐product. To verify the scavenging effect of the nanosheets and NHPH on ROS, 2D‐MnO_2_ nanosheets with different concentrations were incubated in H_2_O_2_ (0.3%) solution and used as catalysts to accelerate H_2_O_2_ decomposition. The hydrogen peroxide detection assay was used to detect H_2_O_2_ after 2D‐MnO_2_ treatment, and the results showed an enhanced ROS scavenging capacity.^[11b]^ Yang et al. developed an injectable hydrogel (Prussian blue nanoparticles@oxidized hyaluronic acid/borax/gelatin,PBNPs@OBG) for IVDD therapy. PBNPs contained within hydrogels have been shown to possess antioxidant capacity. In vitro and in vivo experiments showed that the PBNPs@OBG hydrogel could protect nucleus pulposus cells from excessive ROS production, restore IVD height, and alleviate IVDD.^[^ ^209^ ^]^ Li et al. created GM@CS‐BP microspheres by combining CS‐NPs with encapsulated black phosphorus quantum dots (BPQDs) on GelMA microspheres. These microspheres reduce H_2_O_2_ intensity, stabilize BPQD release, and decrease acid‐sensitive ion channel‐3 and inflammatory factors, promoting nucleus pulposus regeneration and offering a new IVDD treatment approach (Figure 4G).^[^ ^210^ ^]^

#### 8.3.3 Nanocomposite Hydrogels Modulating the Immune Microenvironment

During the process of IVDD, a large number of immune cells infiltrate into the IVD, among which macrophages tend to polarize toward the M1 type, producing a large amount of inflammatory factors that accelerate IVDD.^[^ ^211^ ^]^ Therefore, regulating the immune microenvironment within the IVD to promote macrophage polarization toward the M2 type and reducing the proportion of the M1 type can be considered as a strategy to delay IVDD. Zhao et al. engineered an injectable nanocomposite hydrogel for IVDD therapy. The hydrogel, containing epigallocatechin‐3‐gallate‐coated hydroxyapatite nanorods embedded in *O*‐carboxymethyl chitosan cross‐linked with aldehyde HA, modulated macrophage phenotype to repair IVDD. The hydroxyapatite nanorods promoted ECM anabolism, and epigallocatechin‐3‐gallate induced M2 macrophage polarization to decrease ECM catabolism.^[^ ^212^ ^]^ Similarly, Han et al. developed a composite hydrogel for AF repair by integrating antioxidant, anti‐inflammatory, and cell recruitment properties. The hydrogel, containing mesoporous silica NPs modified by ceria and TGF‐β3, eliminated ROS and induced anti‐inflammatory M2 macrophage polarization. The released TGF‐β3 recruited AF cells and promoted ECM secretion.^[^ ^213^ ^]^ Yang et al. encapsulated MnO_2_ NPs with macrophage membranes overexpressing transmembrane protein TrkA to selectively target macrophages, clearing excessive ROS, and preventing M1 polarization of macrophages.^[^ ^214^ ^]^ Jiang et al. reported that Mg can promote macrophage polarization toward the M2 phenotype, thereby treating IVDD. However, the uncontrolled release and rapid metabolism of Mg are challenges faced in current IVDD treatments. To address this issue, they combined Ca^2+^ and Mg^2+^‐containing silicate ceramics with poly(N‐isopropyl acrylamide) (PNIPAAm) and SA to form a unique injectable hydrogel. Upon injection in vivo, PNIPAAm undergoes sol‐gel transition, forming a protective shell that effectively encapsulates and maintains the released Mg^2+^ at ideal concentrations. These Mg^2+^ ions promote ECM synthesis and immune modulation (upregulating M2/downregulating M1 macrophage phenotype polarization), thereby inhibiting the progression of IVDD.^[^ ^215^ ^]^

#### 8.3.4 Smart‐Responsive Release Nanocomposite Hydrogels for IVDD

The functionality of pH‐responsive hydrogels is grounded in their ability to interact with the surrounding environment through changes in pH. These hydrogels are composed of polymers with ionizable groups, such as carboxylic acids or ammonium salts, which can accept or release protons in response to environmental pH variations.^[^ ^216^ ^]^ This process leads to a change in the polymer's charge state, which in turn influences the hydrogel's volume and structure.^[^ ^217^ ^]^ In an acidic environment, such as that found in IVDD, polyanionic polymers like PAA will ionize, leading to an increase in electrostatic repulsion between the negatively charged groups and consequent swelling of the hydrogel. Conversely, in a more alkaline environment, the ionization is reduced, leading to a decrease in repulsion and a potential shrinkage of the hydrogel.^[^ ^218^ ^]^

**Figure 9:**
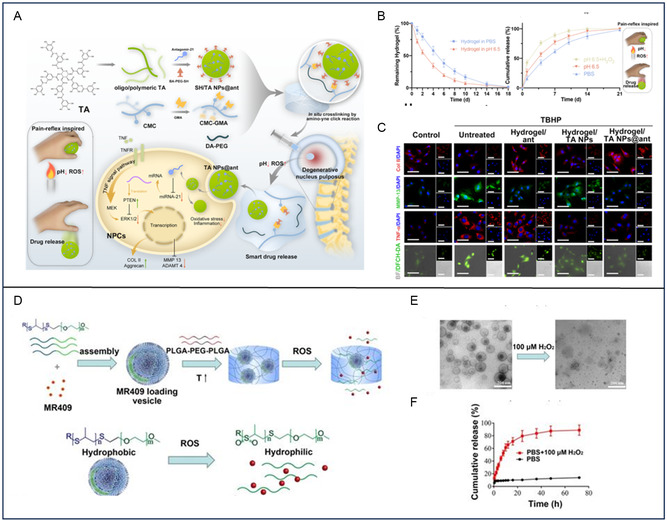
A) Illustration of the design of hydrogels and the mechanisms of IVDD repair promotion. B) The in vitro degradation process of the hydrogel under different conditions suggested that the pathological acidic environment in IVDD accelerates hydrogel collapse and the release result of antagomir‐21 indicated that the inflammatory environment (low‐pH and high‐ROS) promotes antagomir‐21 release from hydrogels. C) immunofluorescence staining. The previous results verified the role of antagomir‐21 in promoting ECM anabolic‐related protein synthesis. Confocal images of DCFH‐DA staining indicated that the hydrogel loaded with TA NPs efficiently eliminated the overproduction of ROS generation in nucleus pulposus cells. Reproduced (Adapted) with permission.^[^ ^223^ ^]^ Copyright 2023, Elsevier. D) Schematic illustration of the thermosensitive hydrogel loaded with ROS‐responsive PPS–PEG vesicles for controlled release of MR409. E) Change in the morphology of MR409‐loaded vesicles in the presence of external H_2_O_2_ (100 μm). F) Cumulative release of MR409 from PPS–PEG vesicles or hydrogel containing vesicles in the presence of 100 μm H_2_O_2_. Reproduced (Adapted) with permission.^[^ ^225^ ^]^ Copyright 2021, Ivyspring International Publisher.

Nanocomposite hydrogels leverage the unique properties of nanomaterials to amplify the pH sensitivity of the hydrogel matrix. For instance, the Ag@MSNs–PAA NPs (mesoporous silica NPs and PAA loaded with metallic silver) mentioned earlier exploit the pH‐responsive swelling and shrinking behavior to control the release of therapeutic agents.^[^ ^219^ ^]^ In the acidic environment of IVDD, these NPs can facilitate a slow and sustained release of drugs, which is critical for reducing inflammation and promoting tissue regeneration.^[^ ^220^ ^]^ The design of pH‐responsive hydrogels can be further refined by incorporating multiscale systems that include nanocomposite materials. These systems, such as polyethylene glycol‐block‐poly(N, N‐diethylaminoethyl methacrylate) (PEG‐b‐PDEAEM) block copolymers with gold nanorods, offer a sophisticated method for drug delivery.^[^ ^221^ ^]^ The release of drugs like doxorubicin (DOX) can be finely tuned to respond to specific pH levels and external triggers like NIR irradiation, allowing for precise control over the timing and rate of drug release in response to the acidic conditions of IVDD. Research has shown that composite hydrogel systems, such as those involving citric acid (CA) cross‐linked with PVA and silver NPs (AgNPs), can provide a sustained and pH‐responsive drug release profile.^[^ ^222^ ^]^ Similarly, Wang et al. designed a novel injectable pH‐responsive hydrogel miRNAs delivery system to locally deliver tannic acid (TA NPs)@antagomir‐21 to IVD core tissues in a two‐phase mechanism: first, inflammation‐triggered on‐demand release from the hydrogel, followed by on‐demand release of TA NPs@antagomir‐21 from the TA NPs@antagomir‐21 in the intracellular delivery of anti‐mir‐21. The hydrogel was mainly composed of glycol methacrylate (GMA)‐modified CMC (CMC–GMA) and was constructed by an amino‐alkyne click reaction, thus it exhibited sensitive pH responsiveness to fulfill the requirements for application in IVDD repair (**Figure** **9**A,B). The hydrogel system exhibits excellent biocompatibility, degradability, and pH responsiveness with tunable gelling time and mechanical strength, and it promotes degenerative medulla repair through inhibition of the MAPK/ERK signaling pathway and reduces inflammation through downregulation of TNF‐α expression (Figure 9C).^[^ ^223^ ^]^ These systems are designed to release therapeutic agents in a controlled manner when exposed to the acidic environment of a degenerated disc, which can help in reducing inflammation and promoting the healing process.

Inflammation‐response hydrogels are being updated with newer iterations. With the gradual advancement of inflammation‐responsive hydrogel research, ROS‐responsive hydrogels have also been developed.^[^ ^224^ ^]^ The design of ROS‐responsive hydrogels can be tailored to achieve specific functionalities. By incorporating chemical moieties that are sensitive to ROS, these hydrogels can be endowed with the ability to respond to the inflammatory microenvironment of IVDD.^[^ ^223^ ^]^

Zheng et al. protected and controlled the release of MR409 by loading it into ROS‐responsive vesicles composed of poly(propylene sulfide)‐poly(ethylene glycol) (PPS–PEG)@oxidized hyaluronic acid/borax/gelatin) amphiphilic polymers, and then encapsulating these vesicle breaks in a thermosensitive PLGA‐PEG‐PLGA hydrogel (Figure 9D). H_2_O_2_ at pathological concentrations accelerated vesicle release as well as disrupted the vesicle structure and facilitated MR409 release (Figure 9E,F).^[^ ^225^ ^]^ In addition to delivering traditional medications, ROS‐responsive hydrogels can encapsulate cells, genes, and other biomaterials, releasing them in response to ROS stimuli to assist in ECM regeneration. GLRX3^+^ extracellular vesicles (EVs‐GLRX3) hydrogels provide an injectable, degradable, and ROS‐responsive hierarchical self‐assembly approach, which has been shown to attenuate mitochondrial damage, mitigate nucleus pulposus senescence, and restore ECM deposition through modulation of redox homeostasis, exhibiting excellent biocompatibility and ability to modulate ROS and MMP.^[^ ^226^ ^]^ Another innovative hydrogel is NHPH, formed by hierarchical self‐assembly of peptide amphiphiles modified with biodegradable 2D nanomaterials, which can continuously release pro‐regenerative cytokines in response to ROS stimulation, effectively inhibit immune responses, and restore regenerative microenvironments in the ECM to facilitate the structural and functional recovery of IVDs after severe injury.^[11b]^

#### 8.3.5 Nanocomposite Hydrogels for Inducing Stem Cell Differentiation and Cell Regeneration

**Figure 10:**
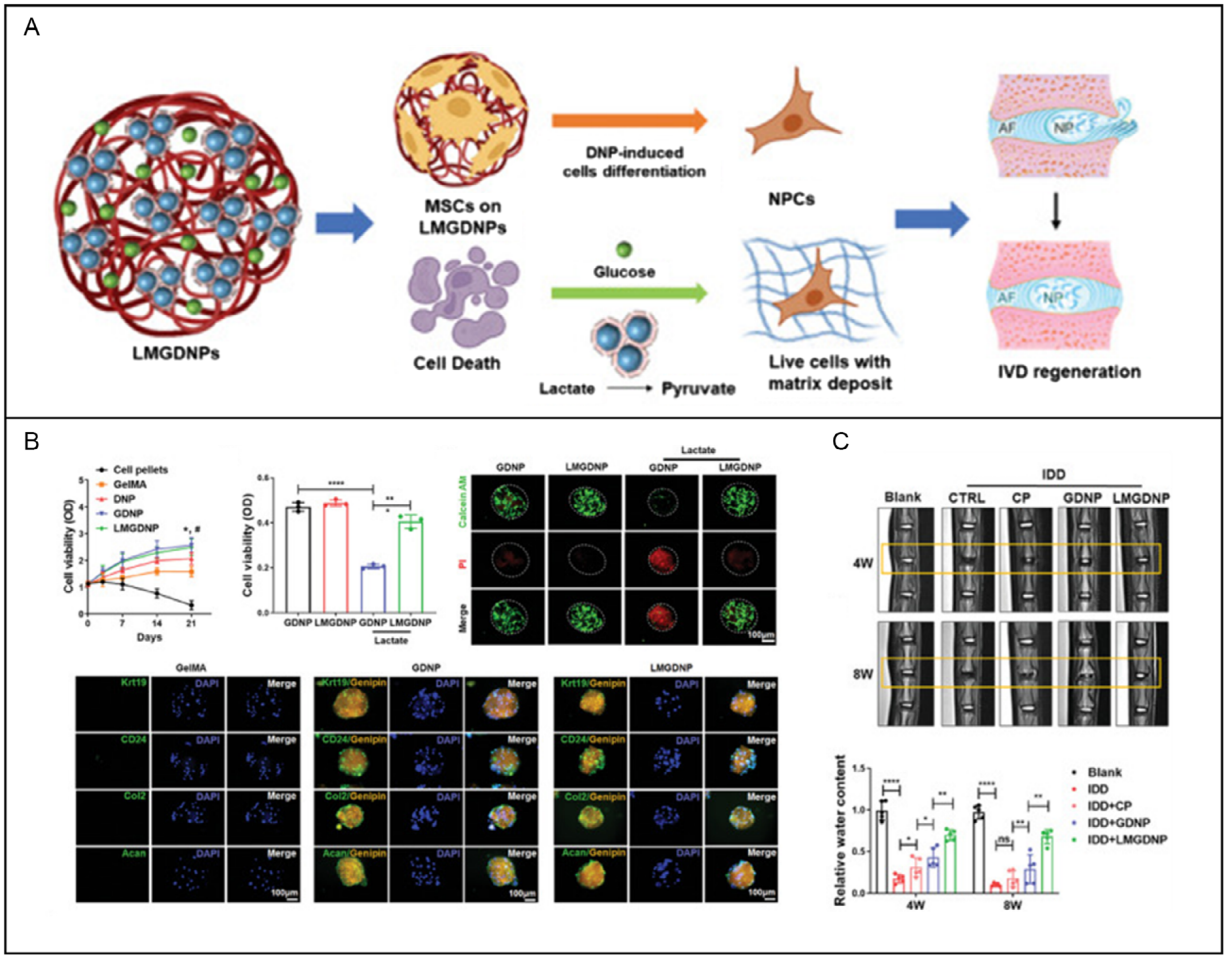
A) Schematic diagram of the effect and mechanism of LMGDNPs in alleviating IVDD. B) Pro‐differentiation capacity and bioactivity of LMGDNPs against lactate. C) Representative images of T2‐weighted magnetic resonance imaging (MRI) minerialized nanoparticle‐loaded GelMA microsphere of rat tails. Boxes indicate the operated disc. Reproduced (Adapted) with permission.^[^ ^231^ ^]^ Copyright 2024, John Wiley and Sons.

As a crucial component of the IVD, the quantity, and vitality of nucleus pulposus cells are closely associated with the process of IVDD. Cell regeneration has proven to be a promising approach for tissue regeneration.^[^ ^227^ ^]^ Wang et al. developed nanostructured gelatin colloidal hydrogels loaded with MSCs for treating IVDD. Gelatin colloidal hydrogel can provide a suitable microenvironment for MSCs to differentiate into nucleus pulposus‐like cells. The chondrogenic markers Sox‐9, Col II, and AGG after coculture, as well as PAX1, a nucleus pulposus‐like cell marker, were significantly upregulated in MSCs cultured in colloidal gels containing chondrogenic medium after 7 and 14 days, respectively.^[^ ^228^ ^]^ Moreover, Gan et al. developed a codelivery system for nucleus pulposus regeneration using MSCs encapsulated in a dextran/gelatin hydrogel with (TGF‐β3‐loaded NPs. The NPs, made of PLGA, released TGF‐β3 gradually, inducing MSC differentiation into nucleus pulposus‐like cells and promoting ECM biosynthesis.^[^ ^229^ ^]^ Lactic acid is a glycolytic product that is found in higher levels in degenerative disc tissues than in blood and other tissues and is detrimental to the activity of MSCs and nucleus pulposus cells.^[^ ^230^ ^]^ Peng et al. performed a cascade decomposition of lactic acid to harmless substances by constructing a lactate oxidase (LOX)‐MnO_2_ nanoenzyme (LM). In addition, glucose‐rich nucleus pulposus matrix hydrogel‐based microspheres (GDNP) were prepared using microfluidics and LM was loaded on GDNP to obtain LOX–MnO_2_ nanoenzymes‐based glucose‐rich nucleus pulposus matrix HMs (LMGDNP) (**Figure** **10**A).^[^ ^231^ ^]^ LMGDNP can induce differentiation of MSCs toward nucleus pulposus cells by breaking down lactate and supplementing it with glucose as a nutrient (Figure 10B). Injecting BMSCs loaded with LMGDNPs into degenerating discs significantly reduces the severity of disc degeneration (Figure 10C), making this an ideal delivery system for regenerative stem cell therapy for IVD.

## 9 Challenges and Future Directions

### 9.1 Challenges in Translating Nanocomposite Hydrogels to Clinical Practice

#### 9.1.1 Scalability and Manufacturing

One of the major challenges in translating nanocomposite hydrogels from the laboratory to clinical practice is the scalability and manufacturing of these complex materials. The production of nanocomposite hydrogels often involves the synthesis and functionalization of NPs, the preparation of polymer solutions, and the precise control of gelation conditions.^[^ ^232^ ^]^ These processes can be time‐consuming, expensive, and difficult to scale up for large‐scale manufacturing. Additionally, the reproducibility and batch‐to‐batch consistency of nanocomposite hydrogels can be challenging to maintain due to the inherent variability in nanomaterial synthesis and the sensitivity of hydrogel properties to slight changes in composition and processing conditions.^[^ ^233^ ^]^ To overcome these challenges, researchers are exploring the use of advanced manufacturing techniques, such as 3D printing^[^ ^66^ ^]^ and microfluidics,^[^ ^234^ ^]^ to enable the scalable and reproducible production of nanocomposite hydrogels with well‐defined architectures and properties.

#### 9.1.2 Long‐Term Safety and Efficacy

Another critical challenge in the clinical translation of nanocomposite hydrogels is the demonstration of their long‐term safety and efficacy in vivo. While many studies have shown promising results in the short term, there is a need for more comprehensive, long‐term studies to evaluate the biocompatibility, biodegradation, and mechanical stability of these materials over extended periods.^[^ ^235^ ^]^ The potential toxicity and immunogenicity of the nanomaterials used in these hydrogels must also be carefully assessed, as some nanomaterials have been shown to elicit adverse biological responses.^[^ ^236^ ^]^ Furthermore, the efficacy of nanocomposite hydrogels in promoting tissue regeneration and functional restoration must be demonstrated in clinically relevant animal models and, ultimately, in human clinical trials.^[^ ^237^ ^]^ The development of standardized, validated preclinical testing protocols and the establishment of clear clinical endpoints will be essential for the successful translation of these materials to clinical practice.^[^ ^238^ ^]^

#### 9.1.3 Regulatory Considerations

The regulatory landscape for nanocomposite hydrogels in bone tissue engineering is complex and evolving, presenting additional challenges for clinical translation. As these materials often combine multiple components, including polymers, nanomaterials, and bioactive molecules, they may be classified as combination products by regulatory agencies, such as the U.S. Food and Drug Administration.^[^ ^239^ ^]^ The regulatory requirements for combination products can be more stringent than those for single‐component materials, necessitating additional testing and documentation to demonstrate safety and efficacy.^[^ ^240^ ^]^ Moreover, the lack of standardized testing methods and quality control procedures for nanomaterials can make it difficult to ensure the consistency and reproducibility of nanocomposite hydrogels across different studies and manufacturing processes.^[^ ^241^ ^]^ Another significant consideration is the cost and availability of hydrogel therapy. The development of a hydrogel from research to clinical translation can cost anywhere from $50 to $800 million.^[^ ^242^ ^]^ This cost will likely translate into high treatment costs for patients and the healthcare system, particularly considering the large number of patients with symptomatic degenerative joint diseases. Another important consideration is the potential for environmental impact.^[^ ^243^ ^]^ The production, use, and disposal of nanomaterials raise concerns about their release into the environment and possible ecological consequences. Therefore, it is crucial to develop sustainable practices for the production and use of nanomaterials in hydrogels. The regulatory landscape for nanomaterials is complex and evolving. Different countries and regions have varying regulations regarding the use of nanomaterials in healthcare products.^[^ ^244^ ^]^ Therefore, researchers and developers must navigate these regulations to ensure compliance and market access for their products.

### 9.2 Future Research Directions

#### 9.2.1 Optimization of Nanocomposite Hydrogel Composition

One of the key future research directions in the field of nanocomposite hydrogels for bone tissue engineering is the optimization of hydrogel composition. While significant progress has been made in the development of nanocomposite hydrogels with improved mechanical properties and bioactive functionalities, there is still a need for further refinement and optimization of these materials to better mimic the complex hierarchical structure and biochemical composition of native bone tissues.^[^ ^245^ ^]^ This will require the systematic investigation of different polymer‐nanomaterial combinations, as well as the fine‐tuning of parameters such as nanomaterial concentration, size, and surface chemistry.^[18b]^ The use of computational modeling and machine learning approaches may help accelerate the design and optimization of nanocomposite hydrogels by enabling the prediction of material properties and biological responses based on hydrogel composition and processing conditions.^[^ ^246^ ^]^

In addition, the adoption of bio‐derived polymers and green synthesis methods is gaining attention as a sustainable approach to hydrogel manufacturing.^[^ ^247^ ^]^ Green synthesis methods, which utilize environmentally friendly processes and non‐toxic reagents, can further reduce the environmental impact of hydrogel production.^[^ ^248^ ^]^ For instance, Gulsonbi et al. developed a biodegradable silver/carboxymethylcellulose‐poly(acrylamide) hydrogel nanocomposite through a green synthesis method using Neem plant extract, which showed promising results for drug delivery applications due to its favorable physicochemical properties and controlled drug release profile.^[^ ^249^ ^]^ Moreover, Pourmadadi et al. utilized green synthesis to develop a pH‐responsive polyvinylpyrrolidone/titanium dioxide hydrogel nanocomposite modified with agarose macromolecules for the sustained and targeted release of the anticancer drug DOX, enhancing its therapeutic efficacy and minimizing side effects.^[^ ^250^ ^]^ Exploring these sustainable materials and methods can lead to the development of nanocomposite hydrogels that are not only effective but also eco‐friendly. Future research should focus on the systematic evaluation of bio‐derived polymers and green synthesis methods to establish standardized protocols for their use in hydrogel manufacturing.^[^ ^251^ ^]^ This includes optimizing the extraction and purification processes of bio‐derived polymers to ensure consistent quality and performance, as well as investigating the long‐term stability and biocompatibility of these materials in vivo.^[^ ^252^ ^]^

#### 9.2.2 Combinatorial Approaches with Other Regenerative Strategies

Another promising future direction for nanocomposite hydrogels in bone tissue engineering is the development of combinatorial approaches that integrate these materials with other regenerative strategies, such as cell therapy, growth factor delivery, and gene therapy.^[^ ^246^ ^]^ The incorporation of stem cells or progenitor cells into nanocomposite hydrogels has been shown to enhance tissue regeneration and integration with the surrounding host tissue.^[^ ^253^ ^]^ Similarly, the controlled release of growth factors and other bioactive molecules from nanocomposite hydrogels can help to guide cell behavior and promote tissue‐specific differentiation.^[^ ^254^ ^]^ Gene therapy approaches, such as the delivery of DNA or RNA NPs encoding for regenerative factors, could also be combined with nanocomposite hydrogels to provide a more sustained and localized therapeutic effect.^[^ ^255^ ^]^ The development of these combinatorial approaches will require close collaboration between materials scientists, biologists, and clinicians to optimize the synergistic effects of the different regenerative components and ensure their safety and efficacy in vivo.

#### 9.2.3 Personalized Medicine Applications

Nanocomposite hydrogels also have significant potential for personalized medicine applications in bone tissue engineering. The ability to tune the mechanical and biological properties of these materials based on individual patient needs and anatomical considerations could enable the development of customized, patient‐specific implants and scaffolds.^[^ ^246^ ^]^ For example, the use of medical imaging data and 3D printing technologies could allow for the fabrication of nanocomposite hydrogels with precise geometries and spatial distributions of nanomaterials and bioactive factors to match the specific defect size and shape in a given patient.^[^ ^256^ ^]^ Additionally, the incorporation of autologous cells or bioactive molecules derived from the patient's tissues could help minimize the risk of immune rejection and enhance the regenerative potential of the implanted material.^[^ ^257^ ^]^ The realization of these personalized medicine applications will require the development of rapid, cost‐effective, and scalable manufacturing processes, as well as the establishment of robust quality control and regulatory frameworks to ensure the safety and efficacy of patient‐specific products.

## 10 Conclusion

In conclusion, nanocomposite hydrogels represent a promising and versatile platform for the treatment of degenerative joint diseases, offering unique combinations of mechanical, biological, and drug delivery properties that can overcome the limitations of current treatment approaches. Despite significant progress in their development, further research and interdisciplinary collaboration are necessary to address remaining challenges, such as optimizing hydrogel composition, understanding long‐term safety and efficacy, and developing scalable manufacturing processes. By addressing these challenges and successfully translating nanocomposite hydrogels from the laboratory to the clinic, we can revolutionize patient care, improve quality of life, reduce healthcare costs, and make a meaningful impact on the lives of millions of patients suffering from debilitating bone conditions worldwide.

## Conflict of Interest

The authors declare no conflict of interest.

## Author Contributions

**Qizhu Chen**, **Zitian Zheng**, and **Mian Lin** drafted and wrote the review. **Zhengyu Guo**, **Hongjie Huang**, and **Qingyun Xue** polished this review. **Shengdan Jiang**, **Jianquan Wang**, and **Aimin Wu** directed and revised this review. **Qizhu Chen**, **Zitian Zheng**, and **Mian Lin** contributed equally to this work.
